# Attenuation of canonical NF‐κB signaling maintains function and stability of human Treg

**DOI:** 10.1111/febs.15361

**Published:** 2020-05-28

**Authors:** Liesa S. Ziegler, Marlene C. Gerner, Ralf L. J. Schmidt, Doris Trapin, Peter Steinberger, Winfried F. Pickl, Christian Sillaber, Gerda Egger, Ilse Schwarzinger, Klaus G. Schmetterer

**Affiliations:** ^1^ Department of Laboratory Medicine Medical University of Vienna Vienna Austria; ^2^ Institute of Immunology Medical University of Vienna Vienna Austria; ^3^ Division of Hematology and Hemostaseology Department of Internal Medicine I Medical University of Vienna Vienna Austria; ^4^ Department of Pathology Medical University of Vienna Vienna Austria; ^5^ Ludwig Boltzmann Institute Applied Diagnostics Vienna Austria

**Keywords:** immune tolerance, NF‐κB signaling, regulatory T cells, TSDR

## Abstract

Nuclear factor ‘κ‐light‐chain‐enhancer’ of activated B cells (NF‐κB) signaling is a signaling pathway used by most immune cells to promote immunostimulatory functions. Recent studies have indicated that regulatory T cells (Treg) differentially integrate TCR‐derived signals, thereby maintaining their suppressive features. However, the role of NF‐κB signaling in the activation of human peripheral blood (PB) Treg has not been fully elucidated so far. We show that the activity of the master transcription factor forkhead box protein 3 (FOXP3) attenuates p65 phosphorylation and nuclear translocation of the NF‐κB proteins p50, p65, and c‐Rel following activation in human Treg. Using pharmacological and genetic inhibition of canonical NF‐κB signaling in FOXP3‐transgenic T cells and PB Treg from healthy donors as well as Treg from a patient with a primary *NFKB1* haploinsufficiency, we validate that Treg activation and suppressive capacity is independent of NF‐κB signaling. Additionally, repression of residual NF‐κB signaling in Treg further enhances interleukin‐10 (IL‐10) production. Blockade of NF‐κB signaling can be exploited for the generation of *in vitro* induced Treg (iTreg) with enhanced suppressive capacity and functional stability. In this respect, dual blockade of mammalian target of rapamycin (mTOR) and NF‐κB signaling was accompanied by enhanced expression of the transcription factors FOXP1 and FOXP3 and demethylation of the Treg‐specific demethylated region compared to iTreg generated under mTOR blockade alone. Thus, we provide first insights into the role of NF‐κB signaling in human Treg. These findings could lead to strategies for the selective manipulation of Treg and the generation of improved iTreg for cellular therapy.

## Graphical abstract

Human regulatory T cells (Treg) differentially integrate extracellular signals (e.g., mTOR, MAP‐kinase, and JAK/STAT signaling) compared to effector T cells. In this work, Ziegler *et al*. for the first time show that Treg also attenuate TCR‐triggered canonical NF‐κB signaling. This unique feature can also be exploited for the generation of inducible Treg. Combination of mTOR and NF‐κB blockade leads to the induction of Treg with increased suppressive capacity and improved functional stability.

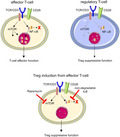

**Abbreviations**
Ala: alanine
AP‐1: activator protein‐1
CCR6: CC chemokine receptor 6
CPD: cell proliferation dye
CTLA‐4: cytotoxic T‐lymphocyte‐associated protein 4
CXCR3: CXC chemokine receptor 3
FOXP: forkhead box protein
GvHD: graft‐versus‐host disease
HEK: human leukocyte antigen‐DR
HLA‐DR: human leukocyte antigen‐DR
ICOS: inducible T‐cell costimulator
IKK: IκB kinase
IL: interleukin
iTreg: induced regulatory T cells
JAK: Janus kinase
MAP‐kinase: mitogen‐activated protein kinase
mTOR: mammalian target of rapamycin
NEMO: NF‐κB essential modulator
NFAT: nuclear factor of activated T cells
NF‐κB: nuclear factor ‘κ‐light‐chain‐enhancer’ of activated B cells
PB: peripheral blood
PD‐1: programmed cell death protein 1
PDK1: phosphoinositide‐dependent kinase‐1
PKCθ: protein kinase C‐θ
qPCR: quantitative PCR
S6RP: S6 ribosomal protein
Ser: serine
SOCS: suppressor of cytokine signaling
STAT: signal transducer and activator of transcription
TCR: T‐cell receptor
Teff: effector T cells
TF: transcription factor
TGF‐β: transforming growth factor‐β
Th17: T‐helper‐17 cell
TIGIT: T‐cell immunoreceptor with Ig and ITIM domains
TNF‐α: tumor necrosis factor‐α
Tr1: type 1 regulatory T cell
Treg: regulatory T cells
Tresp: responder T cells (CPD‐stained, total CD4^+^ T cells)
TSDR: Treg‐specific demethylated region

## Introduction

The immune system is controlled by highly balanced processes that guarantee an appropriate response against pathogens while simultaneously preventing harmful reactions against self‐antigens. Regulatory T cells (Treg) play an important role in maintaining immune homeostasis by mediating tolerance and keeping immune responses in check. Disturbances in this rather small cell population lead to dysregulation of the immune system, causing autoimmune diseases and inflammatory disorders [1]. Stable expression of the transcription factor forkhead box protein 3 (FOXP3) is crucial for Treg phenotype and function. The upregulation of FOXP3 is also seen in CD4^+^ effector T cells (Teff) in a temporary, activation‐dependent manner [2]. Stable FOXP3 expression in functional Treg is maintained by the demethylation of a noncoding sequence on the FOXP3 locus, the so‐called Treg‐specific demethylated region (TSDR), which is constitutively methylated in Teff [3].

Similar to Teff, execution of the immunosuppressive function of Treg is dependent on activation via the triggering of the T‐cell receptor (TCR)/CD3 complex [4], costimulatory and cytokine receptors. Signals from these cell surface molecules are integrated by the initiation of intracellular signaling cascades culminating in the activation of key transcription factors (TFs) such as nuclear factor of activated T cells (NFAT), activator protein‐1 (AP‐1), and nuclear factor ‘κ‐light‐chain‐enhancer’ of activated B cells (NF‐κB). In turn, these TFs activate specific genetic programs that govern effector functions such as proliferation, cytokine production, upregulation of surface markers, and metabolic adaptation. These processes have been defined in numerous studies in Teff. However, the situation in peripheral blood (PB) Treg, which constitute the major Treg subset, has not been fully assessed so far. Previous studies have established that Treg differentially integrate the extracellular cues compared to Teff by attenuating or modifying signal transduction events. In this respect, one key finding was that Treg activation results in attenuated mammalian target of rapamycin (mTOR) signaling [5], although some mTOR activity is needed for Treg function and peripheral homeostasis [6]. This biological principle can be exploited to differentiate and expand Treg from PB CD4^+^ T cells in the presence of the mTOR inhibitor rapamycin (Rapa) [7, 8, 9]. Similarly, recent publications have highlighted that adaptations of intracellular signaling in Treg can be found on various levels including TCR‐proximal signal factors, mitogen‐activated protein kinases (MAP‐kinases), and Janus kinase (JAK)–signal transducer and activator of transcription (STAT) signaling [10, 11].

In this respect, the pathway leading to active NF‐κB signaling is of particular interest due to its crucial importance in the activation of Teff. The NF‐κB family consists of five subunits, which can form homo‐ and heterodimers through their N‐terminal Rel‐homology domain: p65 (RelA), RelB, c‐Rel, p50 (NF‐κB1), and p52 (NF‐κB2) [12]. The most common target of the canonical pathway consists of p65:p50 heterodimers, but also combinations with c‐Rel are possible [13]. In resting cells, the dimers are bound to IκB and are thereby sequestered in the cytoplasm. Upon an activating stimulus, the three IκB kinases (IKK) IKKα, IKKβ, and NF‐κB essential modulator (NEMO) form the IKK complex. This complex mediates the phosphorylation of IκB, which is thereby targeted for degradation. Subsequently, the NF‐κB dimers are released from the inhibitory complex, enter the nucleus, and bind to their target transcription sites [13].

Numerous studies have confirmed the importance of NF‐κB activation for the development of Treg in murine models. Genetic enhancement of NF‐κB signaling induces Foxp3 expression and leads to a higher number of Foxp3^+^ cells [14]. On the other hand, c‐Rel‐deficient mice show massive reduction in Treg numbers [15]. Another group found c‐Rel to be important for Treg development and p65 pivotal for mature Treg identity [16]. In these models, the most affected subsets of c‐Rel ablation were activated Treg residing at tumor sites [17]. Accordingly, melanoma growth can be reduced in mice with c‐Rel deficiency, but not p65 deficiency [17]. Similarly, mice deficient for phosphoinositide‐dependent protein kinase 1 (PDK1), an upstream kinase of the NF‐κB pathway, showed reduced Treg numbers and less suppressive capacity of the existing Treg. However, this effect could not clearly be attributed to NF‐κB, as there are also other downstream targets of PDK1 [18]. In other studies, it was shown that inhibition of protein kinase C‐θ (PKCθ), a downstream target of PDK1 that is important for activation of NF‐κB and other signaling pathways, indeed leads to reduced Treg numbers but confers the remaining Treg with higher suppressive capacity [19, 20].

While the contribution of the NF‐κB pathway to Treg function has thus not been completely defined in mice and reveals a rather ambiguous picture, studies on human Treg are even more scarce. In general, different mutations in the genes for NF‐κB1 and NEMO in patients have been described, which all lead to immunodeficiencies with varying degrees of severity [21, 22, 23]. Patients harboring mutations in the *NFKB1* gene, causing a p50 haploinsufficiency, present with reduced Treg numbers in peripheral blood [24]. Yet, the effects of canonical NF‐κB signaling on the function of human Treg in the periphery have not been investigated at all.

In the last years, Treg have become interesting targets in therapies for cancer, transplantation medicine, and diverse immunological disorders. Understanding the signal integration of cells used in medical therapy is not only helpful for further discoveries but also crucial for appropriate human application. Particularly, the definition of unique signaling patterns in Treg might help to identify novel therapeutic targets for the selective modulation of these cells.

In this study, we therefore investigated the overall role of NF‐κB signaling in human Treg, regarding the functionality of primary human Treg as well as the generation of induced Treg (iTreg) from CD4^+^ T cells using pharmacological and genetic inhibition. We describe that human Treg initiate only attenuated NF‐κB signaling upon activation and blockade of NF‐κB does not affect Treg activation and function. Along those lines, dual blockade of NF‐κB and mTOR signaling enhances function and stability of *in vitro* induced Treg.

## Results

### Human Treg show attenuated canonical NF‐κB signaling following activation

**Fig. 1:**
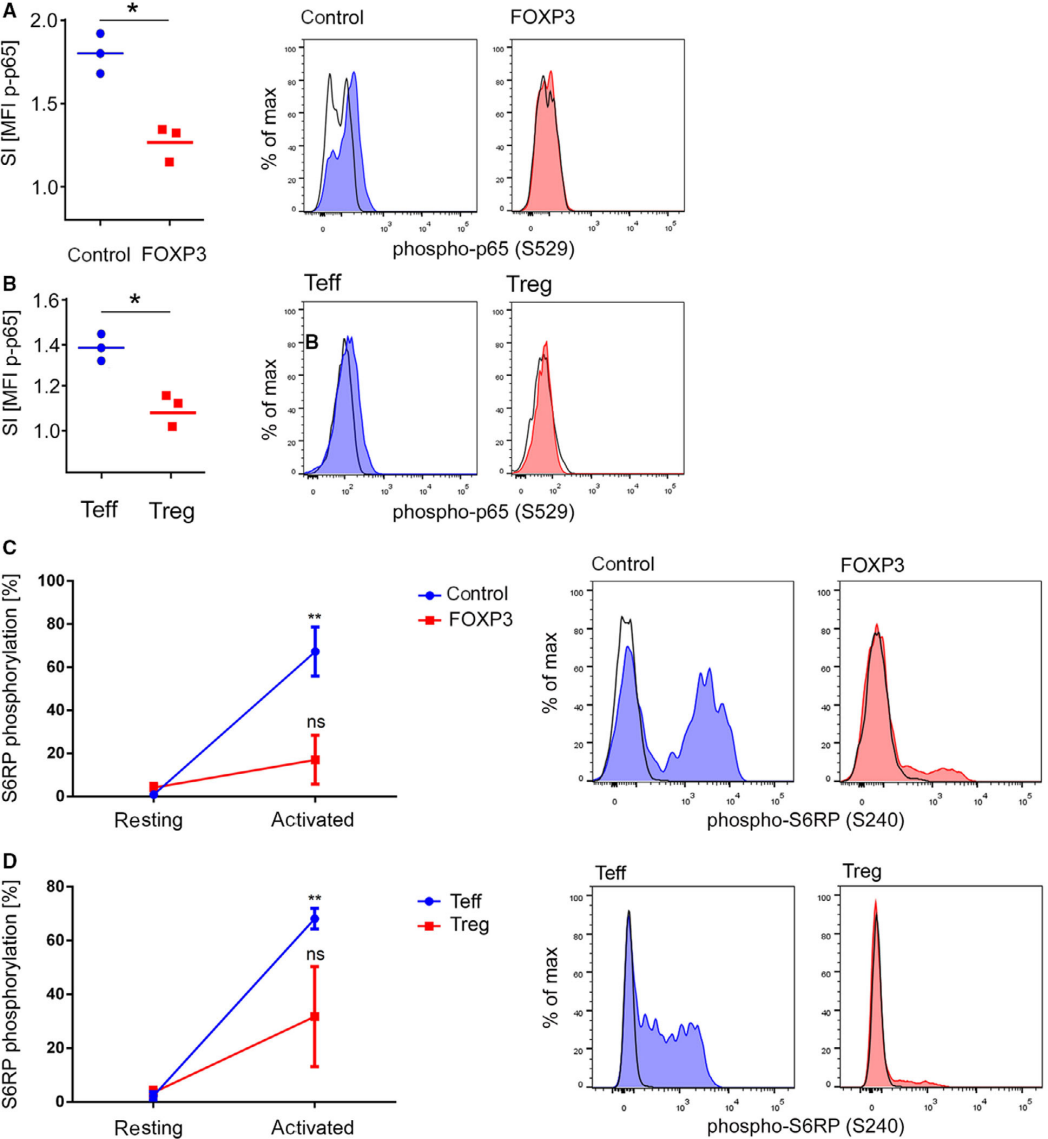
Attenuation of NF‐κB signaling in human Treg. (A) Phosphorylation of p65 on Ser529 was measured via FACS analysis in resting and anti‐CD3/anti‐CD28 activated (24 h) total CD4^+^ T cells transduced with either an empty control vector (blue) or a FOXP3‐encoding vector (red). Left: Statistical analysis of the stimulation index (SI; stimulated : unstimulated cells) of the MFI of phospho‐p65 from three independent donors. *P ≤ 0.05 (paired *t*‐test). (B) Primary Teff (blue) and Treg (red) were isolated from human peripheral blood and activated (24 h), and p65 phosphorylation was measured as in A. Left: Statistical analysis of the SI of the MFI of phospho‐p65 from three independent donors. *P ≤ 0.05 (paired *t*‐test). (A, B) Right panels: Histogram overlay of one representative donor (black line: resting; colored: activated). (C, D) Phosphorylation of S6RP on Ser240 was measured via FACS analysis in resting cells and cells activated for 24 h. (C) Total CD4^+^ T cells were transduced with either an empty control vector (blue) or a FOXP3‐encoding vector (red). Left: Statistical analysis of the percentage of phospho‐S6RP^+^ cells from three independent donors. Phospho‐S6RP levels in activated co‐tg and FOXP3‐tg cells were compared to phospho‐S6RP levels in their resting counterparts, respectively. Data are represented as mean ± SD of the percentage of positive cells; ns = not significant, **P ≤ 0.01 (paired *t*‐test). (C, D) Right: Histogram overlay of one representative donor (black line: resting; colored: activated). (D) Primary Teff (blue) and Treg (red) were isolated from human PB. Left: Statistical analysis of the percentage of phospho‐S6RP^+^ cells from four independent donors. Phospho‐S6RP levels in activated Teff and Treg were compared to phospho‐S6RP levels in their resting counterparts, respectively. Data are represented as mean ± SD of the percentage of positive cells; ns = not significant, **P ≤ 0.01 (paired *t*‐test).

In order to get first insights into NF‐κB signaling in human Treg, total CD4^+^ T cells were transduced with an empty control vector (co‐tg) or with a FOXP3‐encoding vector (FOXP3‐tg), which can be used as a model system to study Treg biology [25, 26]. Subsequently, transgenic cells were isolated by FACS sorting and either activated with anti‐CD3/anti‐CD28 microbeads for 24 h or left unstimulated. In a first set of experiments, phosphorylation of serine (Ser) 529 of the NF‐κB p65 protein, which controls p65 activity [27], was assessed by intracellular flow cytometry. As expected, the abundance of phosphorylated p65 was significantly elevated in co‐tg cells after activation (Fig. 1A). In contrast, phospho‐p65 levels in activated FOXP3‐tg cells remained lower than those in activated co‐tg cells and were not significantly increased compared to their resting counterparts (Fig. 1A). Thus, we gained first indications that NF‐κB signaling differs between activated Teff and Treg.

To confirm this finding in primary human T cells, CD4^+^CD25^low^CD127^high^ Teff and CD4^+^CD25^high^CD127^low^ Treg were FACS‐sorted from human PB mononuclear cells from healthy donors and were activated as above. Again, the levels of phosphorylated p65 were significantly elevated in Teff after activation (Fig. 1B). In accordance with the data from the FOXP3‐tg T cells, significantly attenuated induction of p65 phosphorylation in primary Treg was found following activation (Fig. 1B).

In parallel, we also assessed the phosphorylation status of the mTOR downstream target S6 ribosomal protein (S6RP), since differential activation of the mTOR pathway is well established between Teff and Treg [28]. Thus, quantification of mTOR signaling served as control to validate the specific biology of Treg. Accordingly, we found robust phosphorylation of S6RP in co‐tg and PB Teff, while both FOXP3‐tg and PB Treg displayed significantly reduced mTOR activity (Fig. 1C,D).

**Fig. 2:**
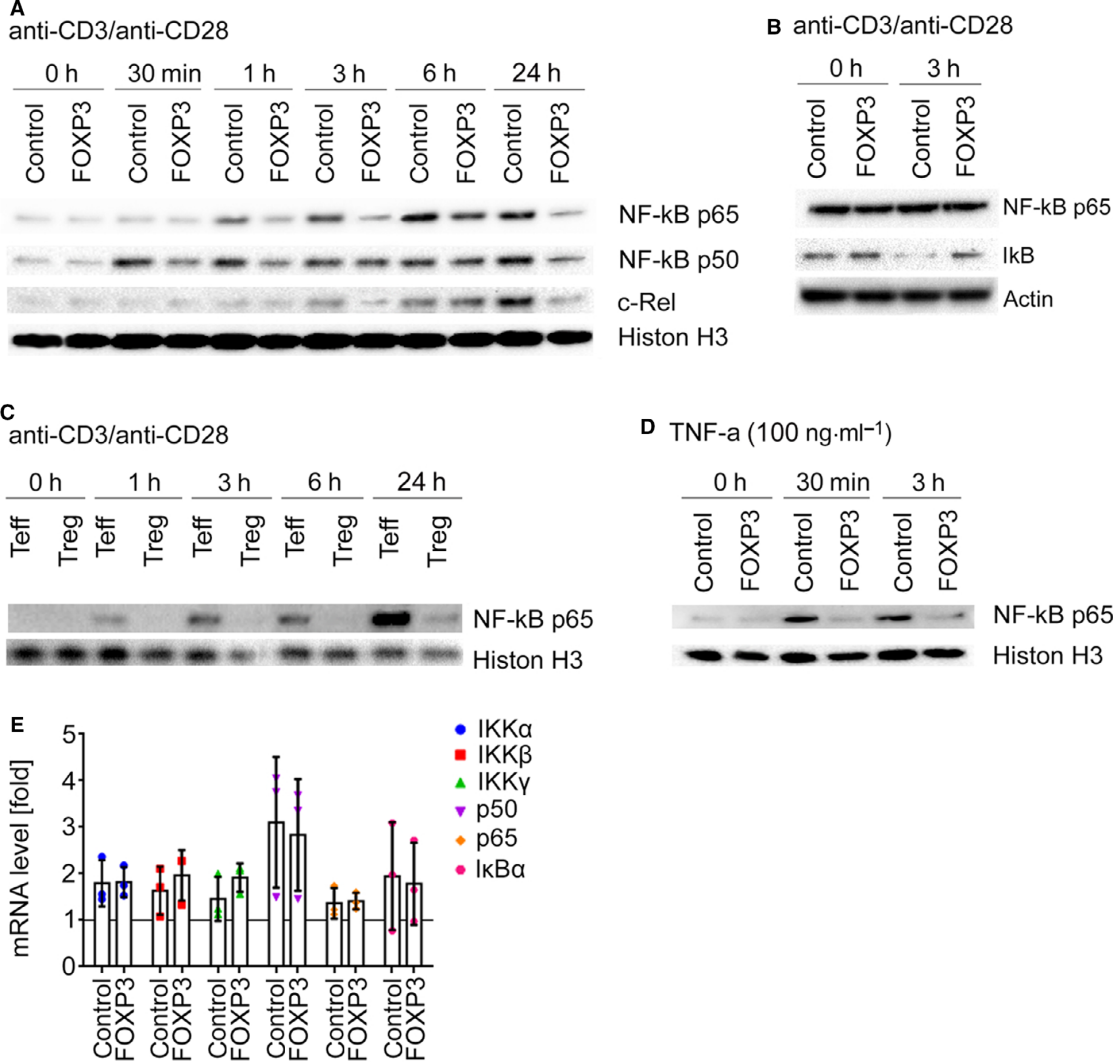
Attenuation of nuclear transport of NF‐κB proteins. (A, B) Total CD4^+^ T cells were transduced with either an empty control vector or a FOXP3‐encoding vector, isolated by FACS sorting, and activated for the indicated time points. (A) Western blot analyses (*n* = 3) of nuclear fractions were performed for NF‐κB p50, p65, and c‐Rel and histone H3 as loading control. (B) Western blot analyses (*n* = 3) of cytoplasmic fractions were performed for NF‐κB p65, IκB, and actin as loading control. (C) Primary Teff and Treg were isolated from human peripheral blood by FACS sorting and activated with aCD3/aCD28 beads for the indicated time points. Western blot analyses (*n* = 3) of the nuclear fractions were performed for NF‐κB p65 and histone as loading control. (D) Western blot analyses (*n* = 3) of nuclear fractions of FOXP3‐transduced or control‐vector‐transduced T cells were performed for NF‐κB p65 and histone H3 as loading control following stimulation of cells with recombinant human TNF‐α for the indicated time points. (A–D) One representative blot per experimental series is depicted. (E) mRNA expression from control‐vector‐transduced or *FOXP3*‐transduced T cells was measured by RT‐PCR. The expression rate of the indicated genes was normalized to ATP‐synthase (*ATP5PB*; reference gene) and resting control‐transduced cells (*n* = 3). Data are represented as mean ± SD (paired *t*‐test).

**Fig. 3:**
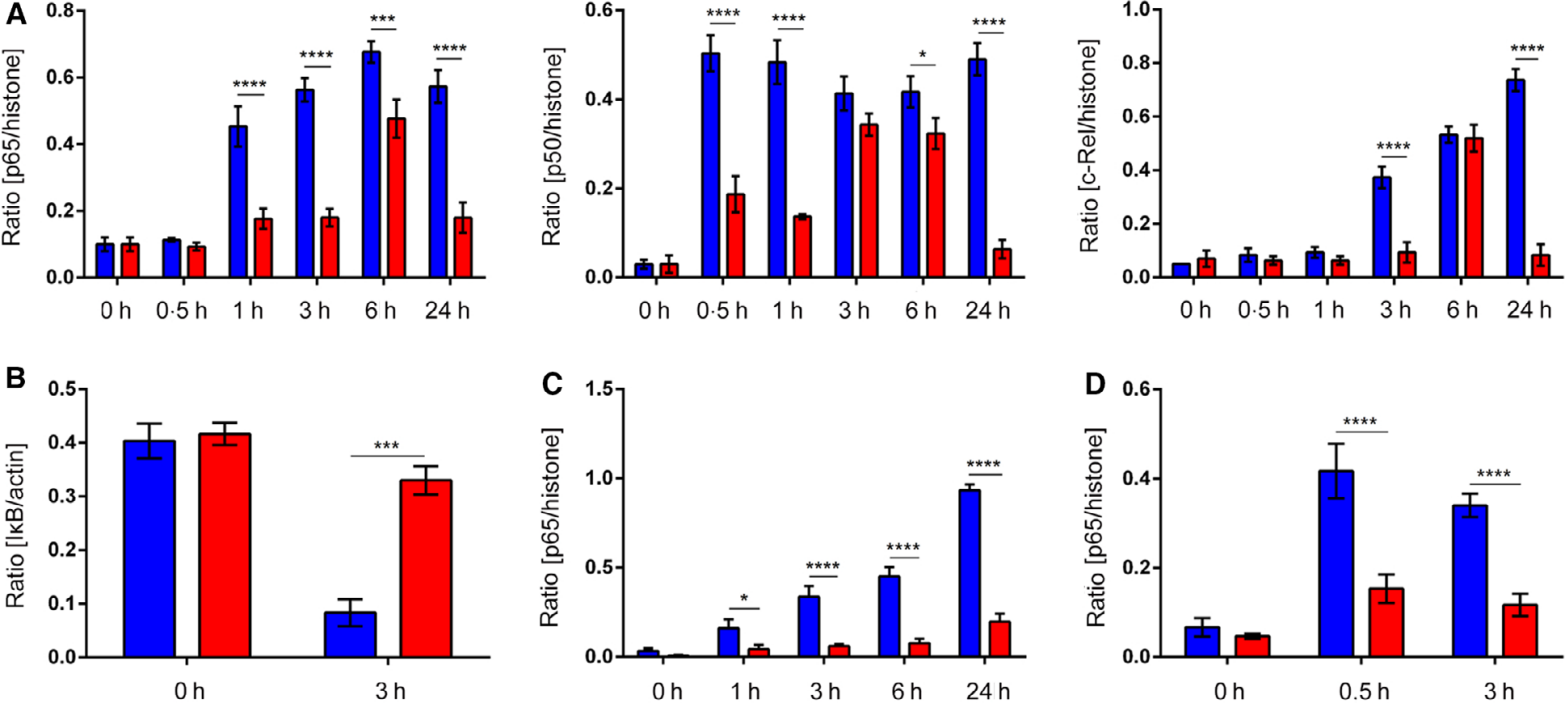
Densitometry of immunoblots shown in Fig. 1. (A, B, D) Total CD4^+^ T cells were transduced with either an empty control vector or a FOXP3‐encoding vector, isolated by FACS sorting, and activated for the indicated time points with (A, B) aCD3/aCD28 beads or (D) 100 ng·mL^−1^ TNF‐α. (C) Primary Teff and Treg were isolated from human peripheral blood by FACS sorting and activated with aCD3/aCD28 beads for the indicated time points. (A, C, D) Western blot analyses (*n* = 3) of nuclear fractions were performed for (A, C, D) NF‐κB p65, (A) p50 and c‐Rel, and (A, C, D) histone H3 as loading control. Density of bands was measured using the ImageJ software, and the ratios of protein : histone density were calculated. (B) Western blot analyses (*n* = 3) of cytoplasmic fractions were performed for NF‐κB p65, IκB, and actin as loading control. (A–D) Data are represented as mean ± SD; *P ≤ 0.1, ***P ≤ 0.001, ****P ≤ 0.0001 (two‐way ANOVA).

Apart from phosphorylation at Ser529, p65 activity is also governed by multiple further phosphorylation sites [29]. Globally, active NF‐κB signaling is marked through nuclear translocation of p65 heterodimers formed with its two partners p50 and c‐Rel. Therefore, we measured nuclear translocation of p50, p65, and c‐Rel in activated co‐tg and FOXP3‐tg T cells in time‐course analyses using immunoblotting of nuclear fractions as a further readout for NF‐κB signaling. These experiments revealed that translocation of all three factors into the nuclei of FOXP3‐tg cells was reduced in comparison with co‐tg T cells (Figs 2, 3 and 2, 3 for statistical analyses of band densitometry). Concordantly, IκB, which is degraded during canonical NF‐κB signaling, was more abundant in the cytoplasm of FOXP3‐tg cells after activation than in co‐tg cells (Figs 2, 3). These results obtained in transgenic cells were subsequently also assessed in primary human CD4^+^CD25^high^CD127^low^ Treg. Similarly to the FOXP3‐transduced T cells, primary Treg showed only minimal translocation of p65 into the nucleus (Figs 2, 3), thus confirming the validity of our experimental approach.

NF‐κB signaling can also be initiated by pro‐inflammatory cytokines such as tumor necrosis factor‐α (TNF‐α). Similar to TCR‐triggered NF‐κB induction, nuclear translocation of p65 in FOXP3‐tg cells was also strongly attenuated in response to recombinant TNF‐α (Figs 2, 3). This suggests that Treg present with a general attenuation of NF‐κB activation irrespective of the upstream signal.

To test whether the reduced NF‐κB signaling in Treg and FOXP3‐tg cells is due to altered expression of one of the pathway components, we analyzed mRNA levels in co‐tg and FOXP3‐tg cells in a resting and activated state using RT‐PCR. Although interindividual differences in mRNA levels of IKKα, IKKβ, IKKγ, p50, p65, and IκB could be observed, no significant differences were found between activated co‐tg and activated FOXP3‐tg cells (Fig. 2E). We consequently conclude that in contrast to Teff, only attenuated activation of NF‐κB signaling is induced in Treg and that this blockade of NF‐κB signaling is not related to different expression levels of the pathway components.

### Blockade of NF‐κB signaling does not affect activation and *in vitro* suppressive capacity of human Treg

**Fig. 4:**
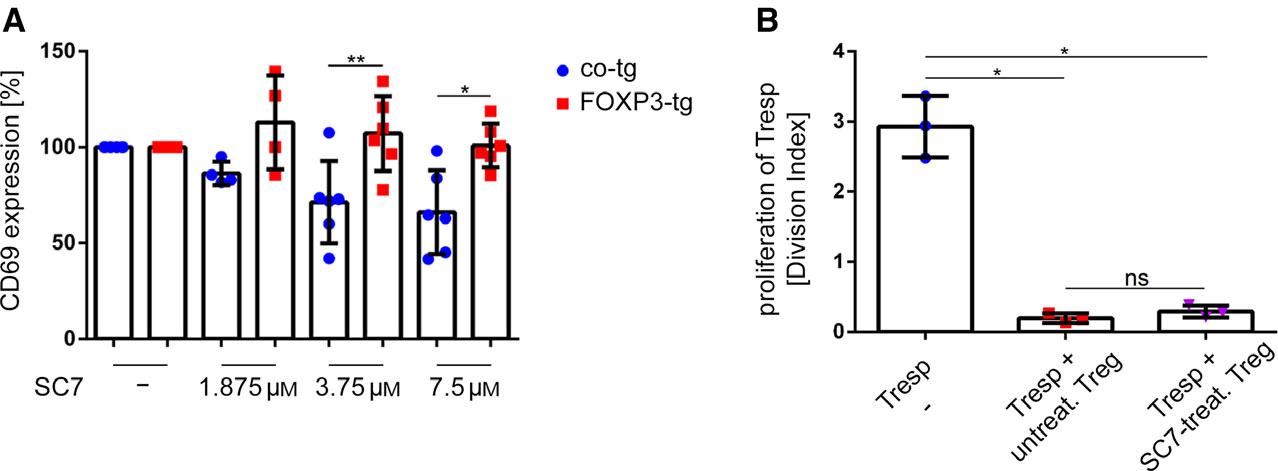
Inhibition of NF‐κB signaling with the small‐molecule inhibitor SC75741 does not affect activation status and suppressive capacity of Treg. (A) Total CD4^+^ T cells from human PB were transduced with either an empty control vector (co‐tg) or a FOXP3‐encoding vector (FOXP3‐tg). Transgenic cells were activated for 24 h in the presence of the indicated concentrations of the NF‐κB inhibitor SC75741 (SC7), and CD69 surface expression was measured via FACS analysis. CD69 expression of co‐tg and FOXP3‐tg cells was normalized to the respective controls activated in the absence of SC7. Data are represented as mean ± SD; *P < 0.05, **P < 0.01 (paired *t*‐test, *n* = 4 for 1.875 µm SC7 concentration, *n* = 6 for all other concentrations). (B) Treg were FACS‐sorted from human PB and pretreated with or without 3.75 µm SC7 for 1 h and then activated with anti‐CD3/anti‐CD28 microbeads. After 4 days, cells were FACS‐sorted for viable cells and cocultured with CPD‐labeled Tresp for another 96 h. Proliferation of Tresp was measured via FACS analysis. Tresp without cocultured cells served as controls. Data are represented as mean ± SD; ns, not significant; *P < 0.05 (one‐way ANOVA, *n* = 3).

The observed attenuation of NF‐κB induction in FOXP3‐tg T cells and PB Treg suggests that basic features of Treg function are largely independent of NF‐κB signaling. To further assess this hypothesis, we activated co‐tg and FOXP3‐tg T cells in the absence or presence of different levels of the small‐molecule NF‐κB inhibitor SC75741 (SC7) [30]. Under these conditions, CD69 upregulation in co‐tg T cells was inhibited in a dose‐dependent manner (Fig. 4A). In stark contrast, FOXP3‐tg T cells were completely unaffected by pharmacological NF‐κB blockade and showed full CD69 upregulation even at highly suppressive SC7 concentrations (Fig. 4A). As a further readout for Treg function, human CD4^+^CD25^high^CD127^low^ Treg were pre‐activated in the absence or presence of SC7 and cocultured with autologous CD4^+^ responder T cells (Tresp) labeled with a fluorescent cell proliferation dye (CPD). Proliferation of these Tresp in coculture was measured as readout for suppressive capacity. Similar to the experiments above, SC7 exposure did not affect the suppressive capacity of the Treg, indicating that NF‐κB signaling is dispensable for this Treg function (Fig. 4B).

**Fig. 5:**
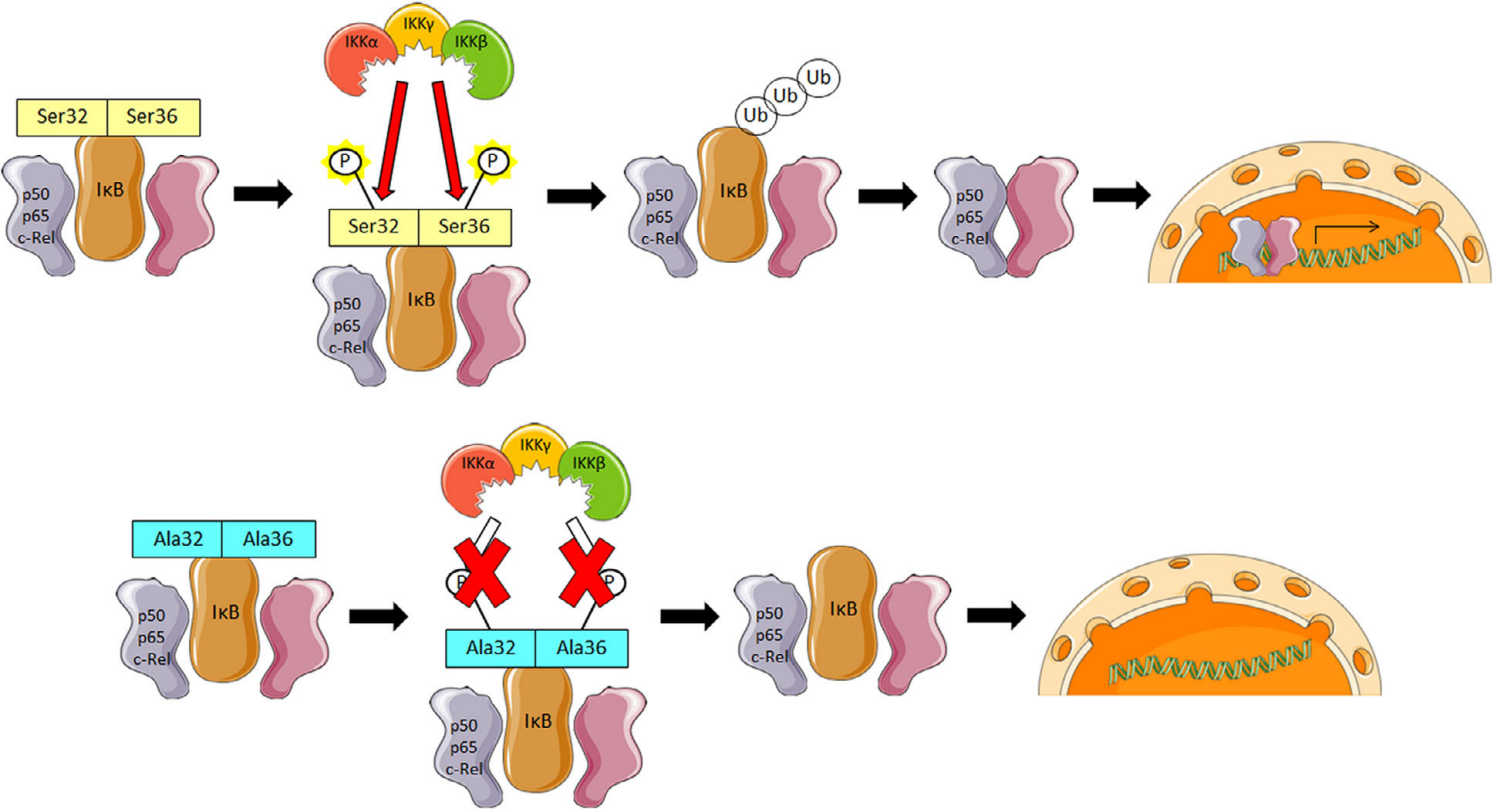
Model of NF‐κB‐repressing mutation of IκB. Serine (Ser) 32 and Ser36 of IκB get phosphorylated during canonical NF‐κB signaling to target it for ubiquitination and following degradation. By using mutagenic primers, bases were changed to code for Ala instead of Ser32 and Ser36. Ala cannot be phosphorylated, and thereby IκB can no longer be targeted for degradation and canonical NF‐κB signaling is disrupted. For this figure, Servier Medical Art templates were used (https://smart.servier.com/).

**Fig. 6:**
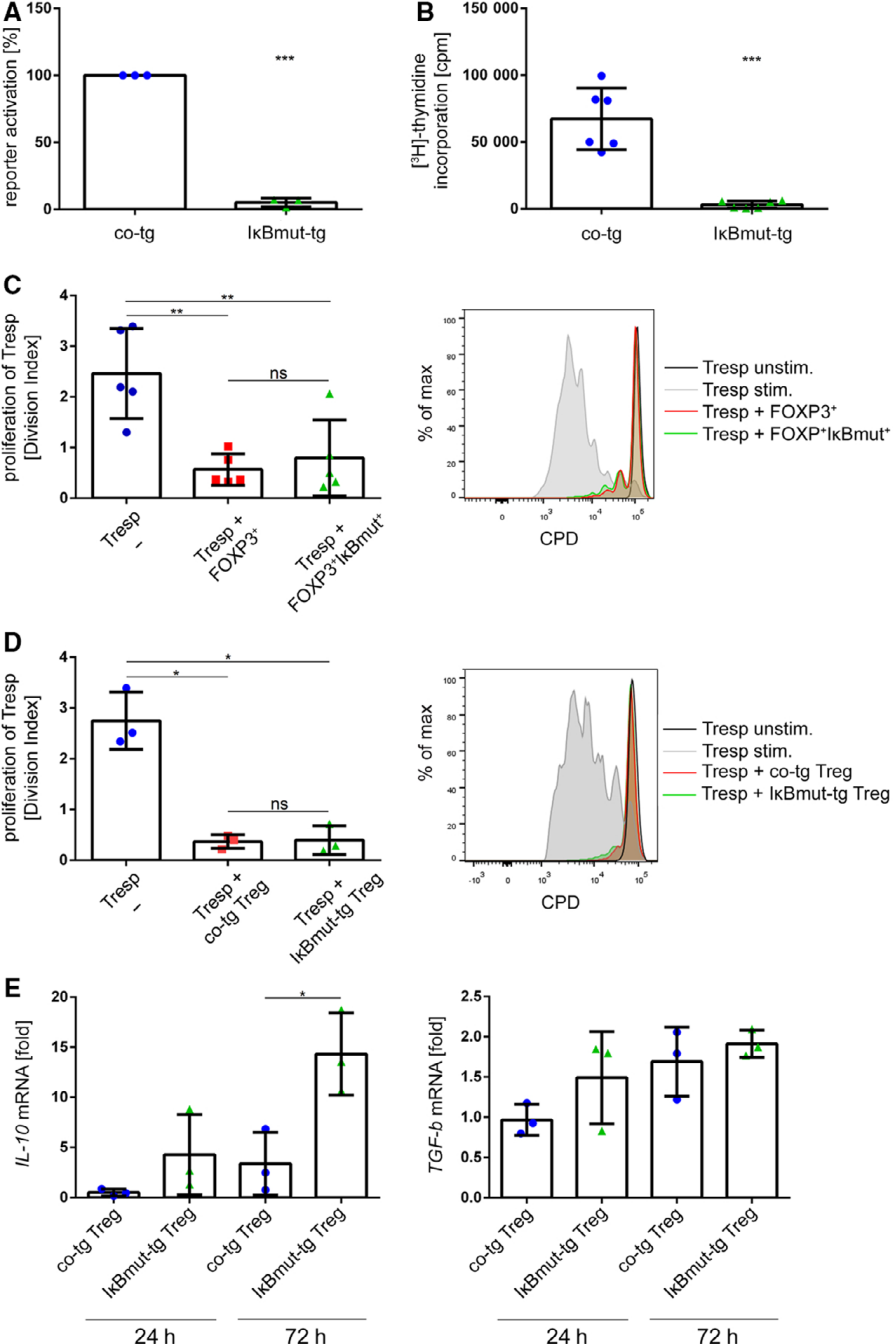
Genetic blockade of NF‐κB signaling does not affect *in vitro* suppressive function of human Treg. (A) A Jurkat reporter cell line harboring an NF‐κB::GFP reporter construct, which allows quantification of NF‐κB promoter activity by GFP expression, was retrovirally transduced with either an empty control vector (blue) or an IRES‐CFP vector coding for a constitutively active IκB construct (IκBmut; green). Cells were activated using anti‐CD3/anti‐CD28 microbeads for 24 h, and GFP fluorescence was measured by flow cytometry. Reporter activation was calculated by normalizing the GFP expression of IκBmut‐tg cells to the GFP expression of co‐tg cells. Data are represented as mean ± SD; ***P < 0.001 (paired *t*‐test, *n* = 3). (B) Total CD4^+^ T cells were transduced with either an empty control vector or a mutagenic IκB construct. Seventy‐two hours after anti‐CD3/anti‐CD28 activation, cells were pulsed with [^3^H]‐thymidine, and after additional 18 h, thymidine incorporation was measured. Data are represented as mean ± SD; ***P < 0.001 (paired *t*‐test, *n* = 6). (C) Total CD4^+^ T cells were transduced either with a FOXP3‐IRES‐GFP vector or cotransduced with a FOXP3‐IRES‐GFP vector in combination with the IκBmut‐IRES‐CFP construct. (D) FACS‐sorted human Treg from PB were transduced with either an empty control vector or the IκBmut construct. (C, D) Cells were FACS‐sorted for GFP/CFP expression, cocultured with CPD‐labeled CD4^+^ Tresp, and activated using agonistic anti‐CD3/anti‐CD28 antibodies. After 96 h, proliferation of Tresp was measured via FACS analysis. (C) Left: Statistical analysis of five independent donors. Data are represented as mean ± SD; ns, not significant; *P ≤ 0.05 (one‐way ANOVA). Right: Histogram overlay of one representative donor. (D) Left: Statistical analysis of three independent donors. Data are represented as mean ± SD; ns, not significant; *P ≤ 0.05 (one‐way ANOVA). Right: Histogram overlay of one representative donor. (E) Control‐vector‐transduced or IκBmut‐IRES‐CFP‐transduced PB Treg were activated using agonistic anti‐CD3/anti‐CD28 antibodies. At the indicated time points, mRNA levels of IL‐10 and TGF‐β were measured by RT‐PCR. Data are depicted as mean ± SD (*n* = 3); *P ≤ 0.05 (one‐way ANOVA).

To further study this feature in Treg, we also aimed to genetically inhibit NF‐κB signaling using a dominant‐negative IκB construct (IκBmut) as described by DiDonato *et al*. [31, 32]. For that purpose, the Ser residues 32 and 36 were mutated into alanine (Ala) residues, thus removing the phosphorylation sites which mediate IκB degradation (Fig. 5). The function of the IκBmut construct was validated in a Jurkat cell line harboring an NF‐κB::GFP reporter construct, which allows quantification of NF‐κB promoter activity by GFP expression [33]. In this system, overexpression of the IκBmut construct completely abrogated reporter activation in the Jurkat reporter cell line compared to Jurkat cells transduced with a control vector (Fig. 6A). To further test the effect in primary human cells, total CD4^+^ T cells were transduced with either a control vector or the IκBmut construct. Again, activation‐induced proliferation of IκBmut‐tg T cells was completely shut off compared to co‐tg T cells, indicating the potency of this approach (Fig. 6B).

Following validation of the IκBmut construct, we investigated its effects on human Treg. In a first set of experiments, we transduced human PB CD4^+^ T cells with the *FOXP3* cDNA in combination with or without the IκBmut construct. As readout for Treg suppressive capacity, transduced transgenic Treg were again cocultured with autologous CPD‐labeled CD4^+^ Tresp. In these experiments, both FOXP3‐tg and FOXP3/IκBmut‐tg cells were able to significantly inhibit proliferation of Tresp with similar potency (Fig. 6C). To verify this finding in primary Treg, we FACS‐sorted CD4^+^CD25^high^CD127^low^ T cells from human PB and also transduced them with either a control vector or the IκBmut construct. As above, coculture assays with CPD‐labeled Tresp were performed. In accordance with the results from the FOXP3‐transgenic T cells, co‐tg Treg and IκBmut‐tg Treg both significantly inhibited proliferation of Tresp (Fig. 6D). Again, the suppressive potency of the transduced Treg was not affected in this setting by the introduction of the IκBmut construct (Fig. 6D). As a further readout for Treg function, we assessed the activation‐induced production of the immunosuppressive cytokines interleukin‐10 (IL‐10) and transforming growth factor‐β (TGF‐β) in co‐tg Treg and IκBmut‐tg Treg. We found massively increased induction of IL‐10 in IκBmut‐tg Treg compared to co‐tg Treg after 72 h of activation. In contrast, the induction kinetics of TGF‐β were not affected by abrogation of NF‐κB signaling (Fig. 6E). Thus, residual NF‐κB induction in Treg does not affect basic suppressive function but selectively downmodulates distinct ‘add‐on’ functions of Treg.

**Fig. 7:**
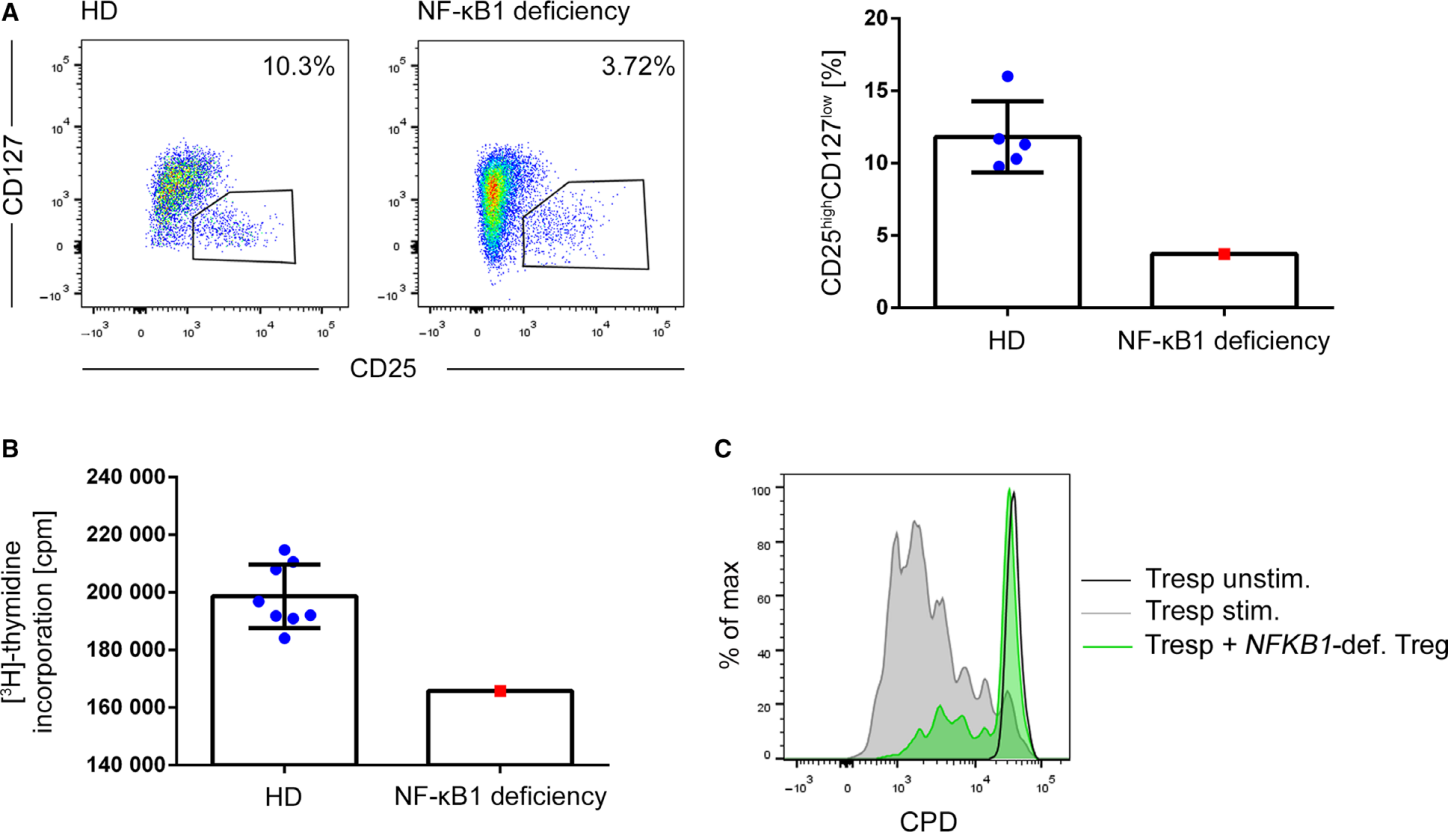
NFKB1 haploinsufficiency does not affect Treg suppressive capacity. (A) FACS staining of PB of an *NFKB1*‐haploinsufficient patient and a sex‐ and age‐matched control cohort of healthy donors. Left: Dot‐plot histograms of the *NFKB1*‐deficient patient and one representative healthy donor (HD) are shown. Right: Bar chart of percentage of CD25^high^CD127^low^ cells of CD4^+^ cells of PB of HD (*n* = 5) and the NF‐κB1‐deficient patient. Data are represented as mean ± SD. (B) CD4^+^CD25^low^CD127^high^ Teff from PB of HDs and one patient with an *NFKB1* haploinsufficiency were FACS‐sorted and activated with anti‐CD3/anti‐CD28 beads for 72 h. Proliferation of Teff was measured by thymidine incorporation. Data are represented as mean ± SD. (C) CD4^+^CD25^high^CD127^low^ Treg of a patient with an *NFKB1* haploinsufficiency were FACS‐sorted and cocultured with CPD‐labeled allogeneic CD4^+^ Tresp. Cells were activated with anti‐CD3/anti‐CD28 beads, and proliferation of Tresp was measured by FACS analysis after 96 h.

**Fig. 8:**
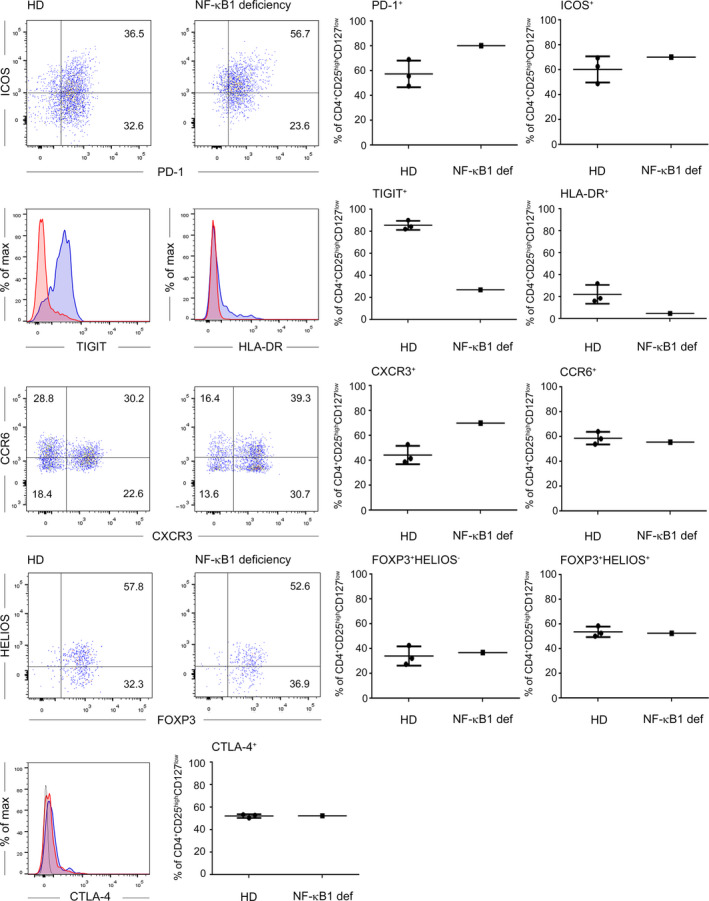
Phenotypic analyses of Treg from an NFKB1‐haploinsufficient patient. FACS staining of PB CD3^+^CD4^+^CD25^high^CD127^low^ of an *NFKB1*‐haploinsufficient patient and sex‐ and age‐matched controls of healthy donors. Left: Dot‐plot and single histograms of the *NFKB1*‐deficient patient (red in histograms) and one representative healthy donor (HD; blue in histograms) are shown. Right: Cumulative data from three HD compared to the *NFKB1*‐deficient patient.

As a further system to study the role of NF‐κB signaling in Treg, we obtained PB specimen from a patient suffering from an *NFKB1* haploinsufficiency. As described for a similar case [24], the percentage of PB Treg was decreased in comparison with age‐ and sex‐matched healthy donors (Fig. 7A). Treg from the patient displayed differential surface expression of programmed cell death protein 1 (PD‐1), T‐cell immunoreceptor with Ig and ITIM domains (TIGIT), human leukocyte antigen‐DR (HLA‐DR), and CXC chemokine receptor 3 (CXCR3) in comparison with age‐ and sex‐matched healthy donors. In contrast, inducible T‐cell costimulator (ICOS) and CC chemokine receptor 6 (CCR6) expression was similar between the patient and the controls. Furthermore, intracellular expression levels of the *bona fide* Treg markers FOXP3, HELIOS, and cytotoxic T‐lymphocyte‐associated protein 4 (CTLA‐4) were similar, indicating that the genetic defect does not compromise the Treg compartment *per se* (Fig. 8). Upon anti‐CD3/anti‐CD28 stimulation, proliferation of CD4^+^ T cells from the patient was significantly decreased in comparison with control donors, validating functional attenuation of NF‐κB signaling in the T‐cell compartment of the patient (Fig. 7B). In accordance with the results from the IκBmut‐tg Treg, FACS‐sorted CD4^+^CD25^high^CD127^low^ Treg from the patient were fully suppressive in coculture experiments with CPD‐labeled Tresp from a healthy third‐party donor (Fig. 7C).

Taken together, these results provide strong evidence that blockade of or genetically diminished NF‐κB signaling does not affect activation and basic suppressive capacity of human Treg, while even increasing production of immunosuppressive cytokines.

### Blockade of NF‐κB signaling during iTreg induction differentially regulates phenotypic markers and enhances FOXP1 expression

**Fig. 9:**
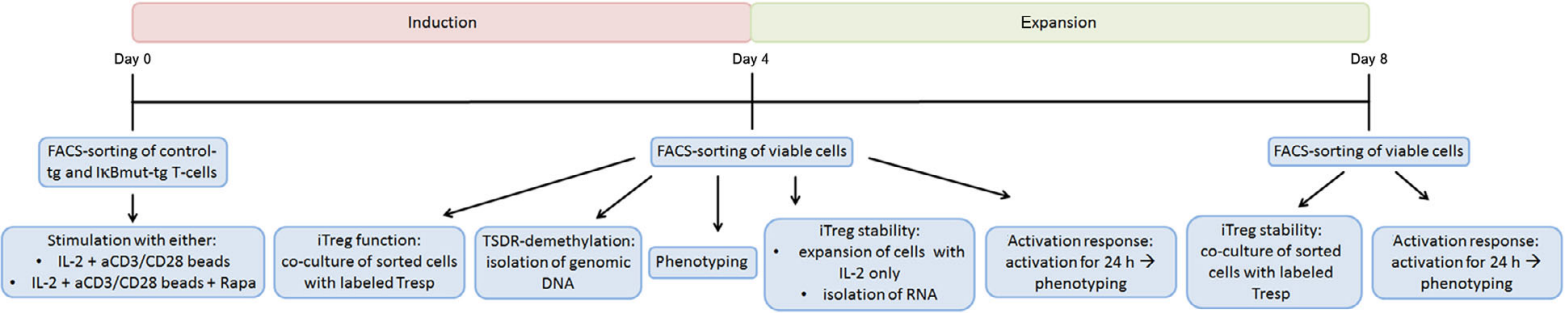
Experimental setup. Total CD4^+^ T cells were transduced with either an empty control vector or a mutagenic IκB construct. On day 0 of the experiment, cells were FACS‐sorted for CFP expression and were activated in the presence of IL‐2 only or in the presence of IL‐2 together with Rapa. After 4 days of polarizing culture conditions, viable cells were isolated by FACS sorting and cells were used for the indicated experimental readouts: For measurement of iTreg function, sorted cells were cocultured with CPD‐labeled Tresp; for measurement of the TSDR demethylation rate, genomic DNA of the sorted cells was isolated and further processed; for iTreg stability assays, sorted cells were partly used for RNA isolation and subsequent RT‐PCR and partly expanded in medium + IL‐2 only for another 4 days without polarizing stimuli. On day 8, viable expanded cells were again isolated by FACS sorting and used for coculture experiments with CPD‐labeled Tresp. On both days 4 and 8, phenotyping of cells was performed and some cells were activated for 24 h and then phenotyped by FACS analysis.

Apart from thymus‐derived Treg, cells with regulatory function can be induced from Teff under tolerogenic conditions or in the presence of the mTOR inhibitor Rapa [7, 8, 9]. Based on the observations described above, we asked whether additional blockade of NF‐κB signaling would modulate this process. For that purpose, we used total PB CD4^+^ T cells and transduced them with a CFP‐encoding control vector or the IκBmut construct. After FACS sorting for CFP expression, cells were activated with anti‐CD3/anti‐CD28 microbeads in the presence of either IL‐2 alone (control culture to assess effectiveness of Treg induction) or IL‐2 plus the mTOR inhibitor Rapa. After 4 days of induction, iTreg were tested for their phenotype, expression of specific transcription factors, the demethylation rate of the TSDR, and their suppressive potential. Some iTreg generated under these different conditions were additionally expanded in IL‐2 only for another 4 days to further assess their functional stability. A chart of the experimental workflow is given in Fig. 9.

**Fig. 10:**
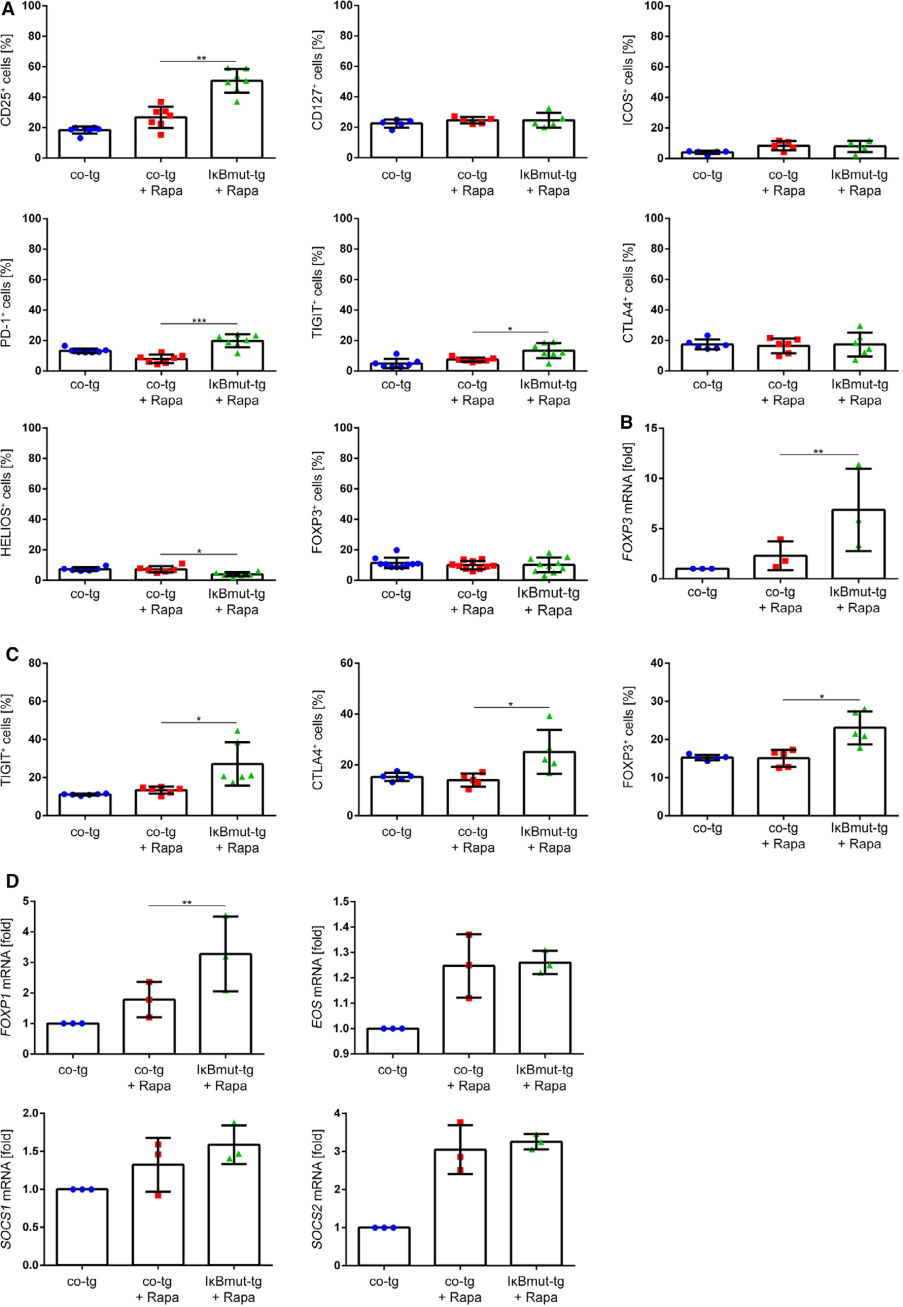
Blockade of NF‐κB signaling during Treg induction modifies iTreg phenotype. (A) On day 4 of iTreg generation, the percentage of cells positive for the Treg‐associated markers CD25, CD127, ICOS, PD‐1, and TIGIT (surface expression) and CTLA‐4, HELIOS, and FOXP3 (intracellular expression) was measured by flow cytometry. Data are represented as mean ± SD; *P ≤ 0.05, **P < 0.01, ***P < 0.001 (one‐way ANOVA). (B) On day 4, RNA was isolated and mRNA levels were measured by RT‐PCR. The expression rate of the *FOXP3* mRNA was normalized to *ATP‐synthase* (*ATP5PB*; reference gene) and resting control‐transduced cells (*n* = 3). Data are represented as mean ± SD; **P ≤ 0.01 (ratio paired *t*‐test). (C) The percentage of TIGIT^+^ cells (surface expression) and CTLA‐4^+^ and FOXP3^+^ cells (intracellular expression) was measured 24 h after activation with agonistic anti‐CD3/anti‐CD28 antibodies. Data are represented as mean ± SD; *P ≤ 0.05 (one‐way ANOVA). (D) mRNA levels of *FOXP1*, *EOS*, *SOCS1*, and *SOCS2* were measured and normalized as in (B). Data are represented as mean ± SD; **P ≤ 0.01 (ratio paired *t*‐test for SOCS1/2; one‐way ANOVA for EOS).

Sole introduction of the IκBmut construct strongly reduced viability of the cultured cells, which did not allow further assessment. However, additional blockade of mTOR rescued the viability of NF‐κB‐inhibited T cells, thus allowing comparison of iTreg generated by Rapa alone or in combination with NF‐κB blockade. Following the above‐described iTreg induction culture, FACS‐based phenotyping of the IL‐2 control cells as well as the co‐tg+Rapa iTreg and IκBmut‐tg+Rapa iTreg was performed. These experiments revealed that NF‐κB blockade significantly upregulated expression levels of the canonical Treg marker CD25 while the surface phenotype of CD127, CD152/CTLA‐4, and ICOS expression was not influenced (Fig. 10A). Furthermore, the immune regulatory markers PD‐1 [34, 35] and TIGIT [36] were significantly upregulated on IκBmut‐tg+Rapa iTreg compared to co‐tg+Rapa iTreg (Fig. 10A). At this time point, intracellular protein levels of the Treg‐associated transcription factor HELIOS were significantly downregulated. Furthermore, FOXP3 protein expression was not altered between the differentially cultured T cells (Fig. 10A). In contrast, *FOXP3* mRNA expression levels were significantly increased under NF‐κB blockade in comparison with control cells and co‐tg+Rapa iTreg (Fig. 10B). This was also reflected by significantly increased FOXP3 protein expression levels in IκBmut‐tg+Rapa iTreg following activation (Fig. 10C). At this time point, also expression of CTLA‐4 and TIGIT was significantly upregulated. Additionally, mRNA levels of the TFs FOXP1 [37] and EOS [38] and the signal modifiers suppressor of cytokine signaling 1 (SOCS1) [39] and SOCS2 [40] were measured, since these factors are involved in Treg function and stability (Fig. 10D). We observed that *FOXP1* mRNA was significantly upregulated in IκBmut‐tg+Rapa iTreg. Expression levels of *EOS*, *SOCS1*, and *SOCS2* were induced in both co‐tg+Rapa iTreg and IκBmut‐tg+Rapa iTreg compared to control cultures, but NF‐κB blockade did not further affect their expression pattern (Fig. 10D). Thus, inhibition of NF‐κB blockade during Rapa‐mediated iTreg polarization selectively affects distinct phenotypic and transcriptional features.

### Blockade of NF‐κB signaling during iTreg induction enhances suppressive capacity, TSDR demethylation, and functional stability

**Fig. 11:**
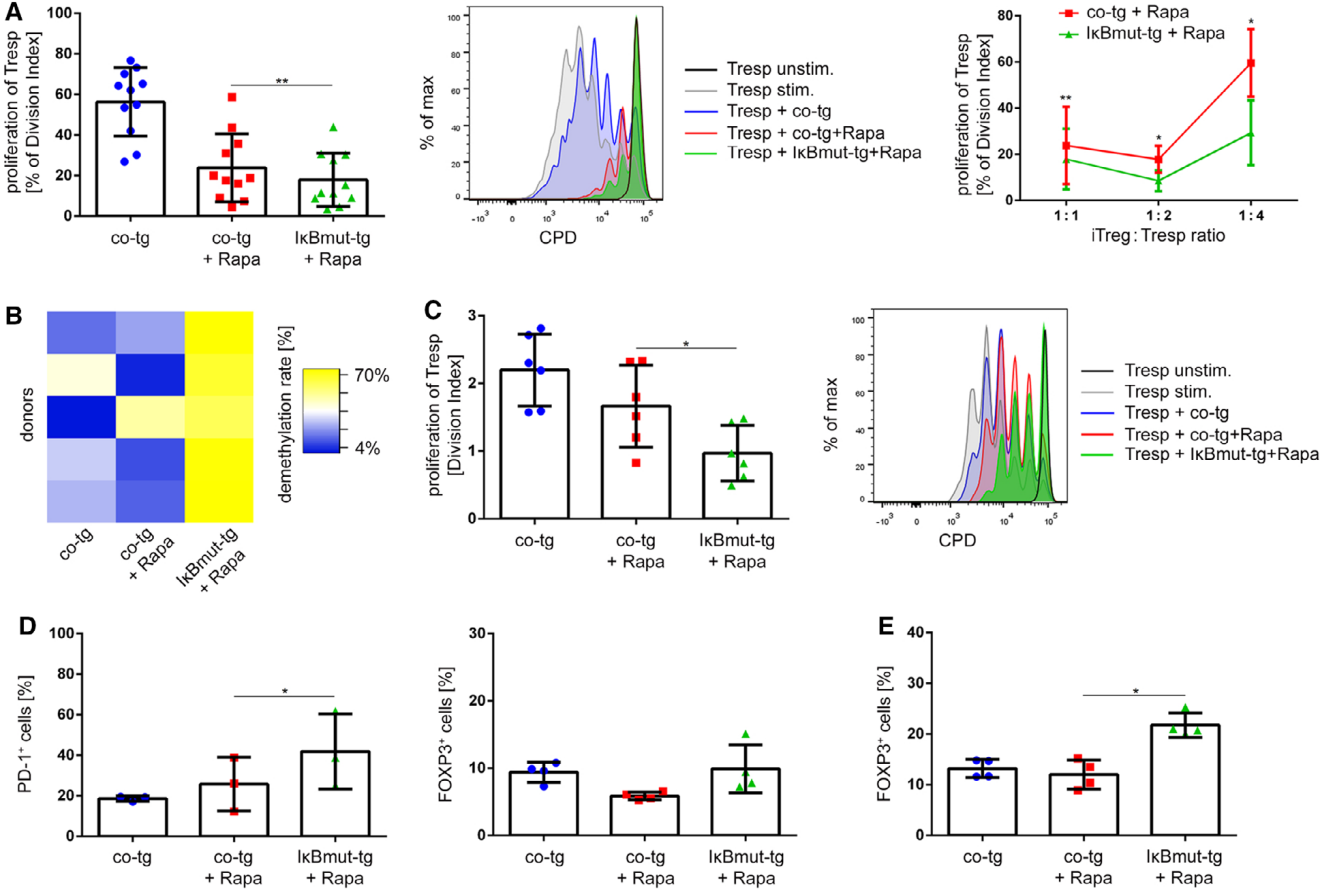
Blockade of NF‐κB signaling during Treg induction increases suppressive capacity of iTreg and enhances their stability. (A–C) After 4 days of polarization of co‐tg and IκBmut‐tg T cells with or without Rapa, viable cells were isolated by FACS sorting and (A) cocultured with CPD‐labeled Tresp and activated using anti‐CD3/anti‐CD28 antibodies. After 96 h, proliferation of Tresp was measured via FACS analysis. Left: Statistical analysis was performed with 11 independent donors from a 1 : 1 ratio of Tresp : iTreg. Data are represented as mean ± SD; *P ≤ 0.05 (one‐way ANOVA). Middle: Histogram overlay of one representative donor. Right: Statistical analyses of cocultures of the respective iTreg at the indicated iTreg : Tresp ratios. (B) Genomic DNA of viable cells was isolated, treated with bisulfite for conversion of demethylated cytosines into uracils, and then used in qPCR with specific primers for unconverted (methylated) and converted (demethylated) TSDR of FOXP3, and the demethylation rate of the indicated samples was calculated according to the formula described in the Materials and methods section. Data are presented as heatmap from five individual donors. The heatmap was generated using Heatmapper [73] (http://www2.heatmapper.ca/). Color code ranges from the lowest measured demethylation rate (4%, blue) to the highest measured demethylation rate (70%, yellow). (C) After 4 days of polarization and an additional 4 days of expansion in only IL‐2 containing medium, cells were FACS‐sorted for viable cells, cocultured with CPD‐labeled Tresp, and activated. After 96 h, proliferation of Tresp was measured via FACS analysis. Left: Statistical analysis was performed with six independent donors. Data are represented as mean ± SD; *P ≤ 0.05 (one‐way ANOVA). Right: Histogram overlay of one representative donor. (D) Percentage of PD‐1^+^ cells (surface expression) and percentage of FOXP3^+^ cells (intracellular expression) in IL‐2 expanded iTreg. (E) Percentage of FOXP3^+^ cells (intracellular expression) following anti‐CD3/anti‐CD28 activation for 24 h. (D, E) Data are represented as mean ± SD; *P ≤ 0.05 (one‐way ANOVA).

To assess the impact of NF‐κB blockade on iTreg function, iTreg from the different induction conditions were cocultured with CPD‐labeled Tresp on day 4 of induction. In these assays, IκBmut‐tg+Rapa iTreg displayed significantly stronger suppressive capacity compared to co‐tg+Rapa iTreg (average suppression of Tresp: co‐tg+Rapa iTreg: 76.14 ± 19.66%, IκBmut‐tg+Rapa iTreg: 82.12 ± 15.07%), which was especially apparent at lower Tresp : iTreg ratios (Fig. 11A). Hence, blockade of NF‐κB signaling during induction of human Treg also enhances their suppressive capacity.

Following the observation that NF‐κB blockade increases expression of *FOXP1*, which is associated with iTreg stability [37], as well as *FOXP3*, we assessed the methylation status of the TSDR, which is strongly demethylated in primary human tTreg [3] but not in Rapa‐induced Treg [41]. Therefore, genomic DNA from the differently induced Treg was isolated on day 4, bisulfite conversion was performed, and the methylation status was determined by specific quantitative PCR (qPCR) [42]. In accordance with the existing literature, induction with Rapa+IL‐2 did not affect the TSDR demethylation in comparison with control cultures in IL‐2 only. In strong contrast, iTreg that were induced by Rapa under additional blockade of NF‐κB signaling showed a significantly higher demethylation rate of the TSDR (Fig. 11B).

As a control for the experimental procedure, CD4^+^CD25^high^CD127^low^ Treg and CD4^+^CD25^low^CD127^high^ Teff were sorted from human PB and the TSDR demethylation was assessed as above. As expected, Teff showed only minimal TSDR demethylation (4–11%), while the TSDR of Treg was nearly fully demethylated (61–98%), verifying the validity of the used method.

To evaluate whether these findings would also translate into enhanced functional stability, iTreg generated under different conditions were expanded with IL‐2 only for another 4 days in the absence of Treg‐inducing stimuli, followed by functional testing (Fig. 11C). Under these conditions, co‐tg+Rapa iTreg largely lost their suppressive capacity. In parallel to the higher TSDR demethylation rate, IκBmut‐tg+Rapa iTreg showed significantly increased suppressive capacity also after 4 days without polarizing supplements (Fig. 11C). Phenotypically, IκBmut‐tg+Rapa iTreg presented with increased PD‐1 expression at this time point, while FOXP3 protein expression was again not different compared to co‐tg+Rapa iTreg (Fig. 11D). As above, activation of the IκBmut‐tg+Rapa iTreg resulted in significant upregulation of FOXP3 expression. Thus, we could show that additional NF‐κB blockade renders Rapa‐induced Treg more stable.

## Discussion

Regulatory T cells constitute a distinct subset of the CD4^+^ T‐cell population, which is marked by distinct phenotypic and functional properties. The core genetic program of CD4^+^ Treg is mediated by the TF FOXP3, which serves as transcriptional activator and repressor and thereby regulates numerous genes involved in Treg‐specific functions [43, 44, 45]. Accordingly, ectopic overexpression of *FOXP3* in murine and human effector T cells largely resembles the properties of PB Treg and can therefore be used as an easily accessible model system to study Treg biology [25, 26]. Already the first seminal Treg studies have established that Treg, similar to Teff, are strictly dependent on TCR‐mediated activation to exert their functions *in vitro* and *in vivo* [46]. Yet, several studies have described that Treg integrate these extracellular signals differently from Teff by modification of intracellular signaling pathways. In this respect, downregulation of mTOR signaling in Treg has been specified as one disparity, and more recently, also differences in TCR zeta chain phosphorylation [10, 47], MAP‐kinase signaling [10], and STAT signaling [10, 11] have been described. NF‐κB constitutes a highly conserved signaling pathway of immune activation which plays crucial roles in virtually all immune cells during their life span from maturation to differentiation to activation [13]. Accordingly, several studies have addressed the role of NF‐κB signaling in Treg biology mainly using knockout mouse models [13, 48]. From these experiments, data have emerged that knockout of the NF‐κB protein c‐Rel impaired Treg development during maturation in the thymus [14, 16, 24, 49]. However, concerning the immunosuppressive function of established Treg, the situation is ambiguous. While some studies have pointed at a role of NF‐κB in Treg activation and suppressive capacity, other authors have found opposing results [18, 19, 20, 50]. In this respect, also differences between knockout of *p65* and *c‐Rel* have become apparent, suggesting a high specificity of differently composed NF‐κB dimers. Yet, the role of NF‐κB signaling in human PB Treg has not been fully addressed so far. We here present definite proof that NF‐κB signaling is attenuated in human PB Treg and typical parameters of activation and suppressive function of human Treg are largely independent of canonical NF‐κB signaling. FOXP3 overexpression in Teff was sufficient to achieve attenuation of NF‐κB signaling, thus suggesting that these features in Treg are driven by FOXP3‐dependent functions. Along those lines, FOXP3‐tg T cells similar to PB Treg present with strongly reduced phosphorylation of NF‐κB p65 and decreased nuclear translocation of the NF‐κB subunits p50, p65, and c‐Rel following activation. Mechanistically, this inhibition of NF‐κB activation was associated with reduced IκB degradation. mRNA levels of major components of the NF‐κB pathway were not altered in FOXP3‐tg T cells. This suggests that stabilization of IκB in Treg is not mediated by direct transcriptional control but either due to the downregulated signaling by upstream kinases or due to a FOXP3‐controlled factor counteracting degradation of phosphorylated IκB. In this respect, also the TNF‐α‐induced activation of NF‐κB was strongly attenuated in FOXP3‐tg T cells. Multiple studies have described roles for TNF‐α in the modulation of Treg function [51]. In light of our observations, it seems likely that these effects are mediated by signaling pathways other than NF‐κB. Thus, these observations warrant further studies to assess the exact molecular mechanism.

For functional studies of NF‐κB in Treg, we resorted to both pharmacological and genetic approaches in FOXP3‐tg and PB Treg. In all systems, we found that activation parameters and the *in vitro* suppressive properties of Treg were not influenced by NF‐κB blockade. Of note, the ectopic introduction of a nondegradable IκB mutant completely abrogated NF‐κB activity in a reporter cell system and activation‐induced proliferation in Teff. Thus, this experimental approach completely inhibits canonical NF‐κB signaling. Given that overexpression of the IκBmut construct in Treg did not affect their function in coculture assays, our data strongly indicate that Treg do not require even minimal levels of canonical NF‐κB signaling for their suppressive capacity *in vitro*. Even further, NF‐κB‐inhibited Treg showed strongly increased production of IL‐10. This gives first indications that the residual NF‐κB activation observed in activated Treg might serve to selectively suppress Treg‐associated features. Our findings might also explain the apparent conundrum that IL‐10 production is not typically observed in Treg assays *in vitro* [46, 52, 53, 54], while numerous studies have described important roles for these cytokines in Treg function *in vivo* [55]. Following our observations, it might be hypothesized that milieu‐specific complete abrogation of NF‐κB signaling in Treg might unleash production of IL‐10 in appropriate situations. It clearly remains to be validated *in vivo* whether this hypothesis holds true and which signals might serve to suppress residual NF‐κB activation in Treg. Furthermore, more in‐depth analyses are warranted to assess which features of Treg are controlled by the level of NF‐κB activation.

Using Treg from an *NFKB1*‐haploinsufficient patient, which were fully suppressive in coculture assays, we further validated the NF‐κB independence of basic Treg features. Combining our results with other studies assessing distinct signaling pathways in Treg [10, 11, 47], the question arises: Which qualitative and quantitative signaling events are required for the activation‐induced functions in human Treg? This topic certainly warrants further investigations both *in vitro* and *in vivo* since it holds the potential to selectively influence Treg function, which could be of therapeutic interest in autoimmunity, transplantation, and tumor immunology.

Treg can be induced from CD4^+^ T cells under tolerogenic conditions. One seminal finding was that *in vitro* T‐cell culture under pharmacological blockade of the mTOR pathway led to differentiation and enrichment of regulatory T cells [7, 8, 9]. These observations were considered as promising to generate large amounts of Treg for adoptive therapy in graft rejection, graft‐versus‐host disease (GvHD), allergy, and autoimmunity. However, the translation into actual clinical settings was impeded by findings that Rapa‐induced Treg could revert to inflammatory T cells once the mTOR blockade was removed [41]. Thus, there is an obvious need to improve the *in vitro* induction regimen especially concerning the stability of the generated Treg. Given the specificity of Rapa for selective inhibition of the mTOR complex, other signaling pathways in these Rapa‐treated T cells rather resemble the situation in Teff and not Treg. This might contribute to the lack of stability in Rapa iTreg. Following our observations in PB Treg, we accordingly hypothesized that NF‐κB blockade could be beneficial in the Rapa‐mediated generation of stable iTreg. To address this issue, we chose the genetic IκBmut overexpression approach described above which guarantees high specificity and efficiency. Phenotypic comparison of co‐tg+Rapa iTreg and IκBmut+Rapa iTreg already revealed first selective effects of additional NF‐κB inhibition. Of note, expression of the hallmark Treg marker CD25 as well as expression of PD‐1 [34, 35] and TIGIT [36], which are associated with increased function of Treg, was significantly induced under dual blockade. The observed phenotypic changes were accompanied by upregulation of the mRNA level of the TF FOXP1 in iTreg generated under mTOR+NF‐κB blockade. FOXP1 has been extensively studied recently, and its expression is indicative of increased stability of murine iTreg *in vitro* and *in vivo* [37]. FOXP1 has dual functions by stabilizing FOXP3 expression as well as physically interacting with the FOXP3 protein [56]. Interestingly, FOXP3 protein expression was not found in Rapa‐induced Treg under the conditions used by us. In this respect, already first seminal studies did not find pronounced effects of Rapa on FOXP3 expression [8, 9] Furthermore, more recent studies show that Rapa exposure even antagonizes the FOXP3‐inducing effect of TGF‐β [57, 58]. Thus, while T cells cultured with Rapa display suppressive capacity, their genetic setup might not fully resemble FOXP3^+^ thymus‐derived Treg, a notion that certainly warrants further investigation. In iTreg generated under NF‐κB/mTOR blockade, FOXP3 protein expression was only significantly upregulated following activation, while mRNA levels were already induced in the basal state. Thus, these cells seem to reside in a preconditioned state that allows them to rapidly acquire Treg features. This finding may also be of general interest since it suggests that FOXP3 protein expression in an unstimulated/resting state is not fully indicative for potential suppressive function of a T cell.

The more Treg‐like phenotype of the iTreg generated under dual blockade was also accompanied by superior suppressive capacity in coculture assays already directly after the induction phase. Furthermore, we assessed the functional stability of the differentially generated iTreg by culturing them for another 4 days in the absence of polarizing stimuli. Under these conditions, iTreg generated under mTOR blockade largely lost their regulatory function. In stark contrast, iTreg generated under dual blockade still exhibited marked suppressive capacity. This was also reflected by the demethylation of the TSDR in the *FOXP3* locus, which is indicative for Treg stability [59, 60]. In parallel to the observations of the functional experiments, mTOR blockade alone did not affect TSDR demethylation in the generated iTreg. However, iTreg generated under NF‐κB/mTOR blockade showed a significantly higher demethylation status at the TSDR, thus more closely reflecting the situation in PB Treg.

Taken together, our studies thus give new insights into the role of canonical NF‐κB signaling in CD4^+^ T cells. We describe for the first time that a distinct subset of CD4^+^ T cells – that is, tTreg – does not require NF‐κB for the initiation of activation‐induced functions. To our knowledge, this is a unique feature of Treg which is not found in any other lymphocyte subset. In this context, it remains to be evaluated whether other FOXP3^−^ Treg subsets, for example, type 1 regulatory T cells (Tr1) [61], also display similar features. Furthermore, Treg can revert to pro‐inflammatory T cells, mostly with a T‐helper‐17 cell (Th17) phenotype, under strongly inflammatory conditions [62, 63, 64, 65]. The question arises whether in this setting, this conversion is at least partly mediated by the re‐acquisition of NF‐κB signaling. Our data also show that NF‐κB strongly counteracts epigenetic and transcriptional changes needed for full Treg identity. Thus, canonical NF‐κB signaling plays a dual role in mature lymphocytes, on the one hand promoting pro‐inflammatory effector immune responses while on the other hand counteracting immunosuppressive mechanisms. It remains to be assessed in preclinical and clinical settings whether these findings can also be confirmed *in vivo*.

In conclusion, we here provide evidence that activation and function of human PB Treg is independent of canonical NF‐κB signaling. This principle can be exploited for the generation of iTreg with enhanced suppressive capacity and stability. This finding could therefore contribute to the selective therapeutic manipulation of Teff and Treg and help to establish novel protocols for the generation of improved iTreg for cellular therapies.

## Materials and methods

### Ethical considerations, cells, and cell culture

The study was approved by the Ethics Committee of the Medical University of Vienna (EC number 1150/2015) and conducted according to the Declaration of Helsinki (1969, including current revisions) of the World Medical Association. Peripheral blood samples of healthy donors were provided by the Austrian Red Cross (Vienna, Austria) upon informed written consent. Blood samples of a healthy donor male cohort (age: 59–74) were obtained upon informed written consent at the Department of Laboratory Medicine, Medical University of Vienna. Peripheral blood mononuclear cells were isolated by standard Ficoll centrifugation. CD4^+^ T cells were isolated using the MagniSort Human CD4 T cell Enrichment Kit (Invitrogen/Thermo Fisher Scientific, Waltham, MA, USA) according to the manufacturer's protocol. Purity was assessed by flow cytometric analyses using monoclonal antibodies against human CD3 (clone SK7; eBioscience/Thermo Fisher Scientific) and against human CD4 (clone SK3; eBioscience, San Diego, CA, USA) and was found to be above 95%. All functional assays were performed in IMDM+GlutaMax (Gibco/Thermo Fisher Scientific) supplemented with 10% fetal bovine serum (Gibco, Carlsbad, CA, USA), 10 µg·mL^−1^ gentamicin (Gibco), and 1.25 µg·mL^−1^ amphotericin B (Lonza, Basel, Switzerland).

A Jurkat reporter cell line harboring an NF‐κB::GFP reporter construct, which allows quantification of NF‐κB promoter activity by GFP expression [33], was cultured in IMDM+GlutaMax as described above.

Human embryonic kidney (HEK) 293 cells (ATCC, Manassas, VA, USA) were cultured in DMEM+GlutaMax (Gibco) supplemented with 10% fetal bovine serum (Gibco), 10 µg·mL^−1^ gentamicin (Gibco), and 1.25 µg·mL^−1^ amphotericin B (Lonza).

### Recruitment and clinical course of an NFKB1‐haploinsufficient patient

A 58‐year‐old male patient with a clinical course of CVID due to an *NFKB1* haploinsufficiency was recruited at the outpatient ward of the Division of Hematology and Hemostaseology of the Medical University of Vienna. First, diagnosis of late‐onset agammaglobulinemia (IgG 238 mg·dL^−1^; IgA < 33 mg·dL^−1^; IgM 43 mg·dL^−1^) was made in 2004. The patient remained clinically asymptomatic with low immunoglobulin levels until 2017 when he showed increased frequency of bacterial infects. Subsequently, immunoglobulin substitution led to an increased clinical improvement with a reduction of infectious disease episodes. In 2018, whole‐exome sequencing revealed an autosomal‐dominant heterozygous mutation in the *NFKB1* gene (c.259‐1G>C) leading to a defective splice site at the 3′ end of exon 5. At the time of recruitment, the patient presented without infection and did not take any medication.

### Cloning of a constitutively active IκB mutant construct (IκBmut)

In order to generate a nondegradable IκBmut construct, the two serine residues Ser32 and Ser36 were replaced with Ala as described before [31, 32].

The cDNA encoding human IκB was amplified from a human T‐cell library [66], and PCR mutagenesis was performed using the following primers (HindIII and NotI restriction sites are underlined, and mutant Ala residues are bolded):

IκB for: 5′ gcgcccaagcttgccaccatgttccaggcggccgagcg 3′
IκB rev: 5′ ggcgcgcggccgctttataacgtcagacgctggcctcca 3′
IκB mut for: 5′ gaccgccacgac**gcc**ggcctggac**gcc**atgaaagacga 3′
IκB mut rev: 5′ tcgtctttcat**ggc**gtccaggcc**ggc**gtcgtggcggtc 3′.

The cDNA was then cloned into the pMMP‐IRES‐CFP vector using the restriction enzymes *HindIII* and *NotI* (both Thermo Fisher Scientific).

### Retroviral overexpression

The used pMMP‐FOXP3‐IRES‐GFP/pMMP‐FOXP3‐IRES‐CFP and empty control pMMP‐IRES‐GFP/CFP vectors were described elsewhere [67, 68]. For transfection of HEK cells, the Ca_2_PO_4_ method was used as described previously [69]. In short, 10 µg pMD‐MoMLV gag‐pol, 5 µg pMD‐GalV, and 15 µg transgene cDNA in the pMMP‐IRES‐GFP/CFP vector were diluted in 900 µL ddH_2_O, and 100 µL 2.5 m CaCl_2_ was added and incubated for 5 min. Subsequently, 1ml HEPES‐buffered saline (Sigma‐Aldrich, St. Louis, MO, USA) was added, followed by incubation for 5 min. Afterward, the mixture was spread on HEK cells at 10% confluency. Twenty‐four hours later, HEK cells were supplemented with fresh media and cultured for another 48 h to allow virus accumulation in the supernatant. At this time point, cell‐free supernatant was harvested and either used directly for T‐cell transduction or frozen at −80 °C.

For transduction, T cells (5 × 10^6^/well) were stimulated with anti‐CD3/CD28‐coated microbeads (Dynabeads, Invitrogen; anti‐CD28, BD, Franklin Lakes, NJ, USA; anti‐CD3, OKT3, Thermo Fisher Scientific) in a 2 : 1 cell‐to‐bead ratio and 300 U·mL^−1^ IL‐2 (PeproTech, London, UK). Cell‐free retroviral supernatant from HEK cells was added to T cells 48 h after stimulation and centrifuged at 900 ***g*** for 90 min in the presence of 8 µg·mL^−1^ polybrene (Sigma‐Aldrich). On the next day, cells were supplemented with fresh IMDM and rested for another 4 days.

### Flow cytometry

For all flow cytometric analyses, all washing steps were performed in PBS (Gibco) + 0.5% FBS (Gibco) + 0.05% sodium azide (Sigma‐Aldrich). Cells were stained with eFluor450‐, eFluor506‐, FITC‐, PE‐, APC‐, PerCP‐Cy5.5‐, PE‐Cy7‐, or APC‐Cy7‐conjugated human monoclonal antibodies against CD3 (clone: SK7), CD4 (clone: SK3), CD25 (clone: BC96), CD69 (clone: FN50), CD127 (clone: eBioRDR5), TIGIT (clone: MBSA43), PD‐1 (clone: eBioJ105), ICOS (clone: C398.4A) (all Thermo Fisher Scientific), and mouse isotype controls, incubated for 30 min at 4 °C, and then washed once.

For whole‐blood staining, EDTA‐anticoagulated blood was stained with the above‐described antibodies for 15 min at room temperature in the dark, and then, 2 mL of BD Pharm Lyse buffer (BD) was added, and it was again left in the dark at room temperature for another 15 min. Cells then were washed once.

For intracellular staining, cells were fixed and permeabilized using the eBioscience Foxp3/Transcription Factor Staining Buffer Set (Invitrogen/Thermo Fisher Scientific) according to the manufacturers' instructions. Permeabilized cells were stained with PE‐, APC‐, PE‐Cy7‐, or APC‐Cy7‐conjugated human monoclonal antibodies against FOXP3 (clone: PCH101), CTLA‐4 (clone: 14D3), and HELIOS (clone: 22F6) (all Thermo Fisher Scientific). Cells were analyzed on a FACSCanto II cytometer (BD).

For Phosflow™ stainings, cells were harvested and fixated with 100 µL prewarmed BD Phosflow™ Fix Buffer I (BD) for 10 min at 37 °C. After centrifugation, cells were permeabilized with 300 µL prechilled 90% methanol for 30 min at −80 °C. Then, cells were washed three times and antibodies against phospho‐p65 (pS529, clone: K10‐895.12.50; BD) and phospho‐S6RP (pS240, clone: N4‐41; BD) were added, and the cells were incubated for 1 h at room temperature in the dark. Cells were washed once before analysis. All flow cytometry data were analyzed using flowjo software (version 10; Tree Star, Ashland, OR, USA).

For cell sorting, cells were washed in PBS + 0.5% FBS + 2 mm EDTA (Sigma‐Aldrich). Cells were FACS‐sorted on a FACSAria Fusion cell sorter (BD). Teff were identified by the CD4^+^CD25^low^CD127^high^ phenotype, while Treg were identified by the CD4^+^CD25^high^CD127^low^ phenotype.

### Jurkat reporter assays

A Jurkat reporter cell line expressing a GFP reporter gene under control of a minimal NF‐κB promoter [33] was transduced to overexpress either an empty control‐IRES‐CFP vector or the IκBmut‐IRES‐CFP construct. Subsequently, CFP^+^ cells were isolated by FACS sorting and 2 × 10^5^ cells were stimulated with anti‐CD3/anti‐CD28 microbeads at the indicated cell‐to‐bead ratios. Twenty‐four hours after activation, Jurkat cells were analyzed by flow cytometry for GFP reporter expression as a readout for NF‐κB promoter activity.

### RT‐PCR

RNA was isolated from 2 × 10^5^ control‐ or FOXP3‐transduced, resting or activated (cells : beads = 2 : 1) T cells using the RNeasy Micro Kit (Qiagen, Hilden, Germany), and cDNA was generated by reverse transcription with random hexamer primers. Transcriptional levels were quantified using the Luna^®^ Universal qPCR Master Mix (New England Biolabs, Ipswich, MA, USA) on a 7900HT Fast Real‐Time PCR system (Applied Biosystems, Foster City, CA, USA) and set relative to the transcriptional levels of ATP‐synthase peripheral stalk‐membrane subunit 5b, used as reference. The following primers were used:

ATP‐synthase for: 5′ gcgtcgaaaggaacaagaac 3′
ATP‐synthase rev: 5′ ctccttttcctgctgtgtgg 3′
IKKα for: 5′ gggaacgtctgtctgtacca 3′
IKKα rev: 5′ tggcaccatcgttctctgtt 3′
IKKβ for: 5′ gctggatcagggcagtcttt 3′
IKKβ rev: 5′ tccagtagtcgacggtcact 3′
IKKγ for: 5′ ctgaagaggcagaaggagca 3′
IKKγ rev: 5′ gtcacctgggctttcacaga 3′
p50 for: 5′ tggactacctggtgcctcta 3′
p50 rev: 5′ tccatttgtgaccaactgaaca 3′
p65 for: 5′ ggagcaggctatcagtcagc 3′
p65 rev: 5′ agagccgcacagcattcag 3′
IκBα for: 5′ gagccctggaagcagcag 3′
IκBα rev: 5′ cttcacctggcggatcactt 3′
FOXP1 for: 5′ gtcagccatgaacggatgga 3′
FOXP1 rev: 5′ agccataaaaagcctggggt 3′
FOXP3 for: 5′ aacatgcgaccccctttc 3′
FOXP3 rev: 5′ attgagtgtccgctgcttct 3′
SOCS1 for: 5′ cacccccggacgctatgg 3′
SOCS1 rev: 5′ ctctgctgctgtggagactg 3′
SOCS2 for: 5′ gaatggcggggaagggac 3′
SOCS2 rev: 5′ aggtagtctgaatgcgagct 3′
IL‐10 for: 5′ gcctaacatgcttcgagatc 3′
IL‐10 rev: 5′ tgatgtctgggtcttggttc 3′
TGF‐β for: 5′ ggctaccatgccaacttctg 3′
TGF‐β rev: 5′ cgggttatgctggttgta 3′
EOS for: 5′ gcggcatggtctgtattgga 3′
EOS rev: 5′ cttctgggtgaaggaggcac 3′

For relative expression levels, ΔCt values (ΔCt = Ct_gene_ − Ct_ATP‐synthase_) were calculated and normalized to resting control‐transduced T cells. Fold expression was calculated according to the formula $2^{\Delta {\text{Ct}}_{\text{normalized}}}$.

### Proliferation assay

For measuring proliferation, 1 × 10^5^ co‐tg and IκBmut‐tg CD4^+^ T cells were stimulated with anti‐CD3/anti‐CD28 microbeads (cells : beads = 2 : 1). Seventy‐two hours after activation, cells were pulsed with [^3^H]‐thymidine (1 µCi per well; PerkinElmer, Boston, MA, USA) and cultured for another 18 h before being analyzed on a Packard scintillation counter (Packard, Meriden, CT, USA).

### Western blotting

At the indicated time points, cells (either unstimulated, activated with aCD3/aCD28 beads, or activated with recombinant human TNF‐α, 100 ng·mL^−1^; PeproTech) were separated into nuclear and cytoplasmic fractions using the NE‐PER™ Nuclear and Cytoplasmic Extraction Kit (Thermo Fisher Scientific), with the following modifications: used CERI per sample: 50 µL + phosphatase inhibitor 1 : 25 (Sigma‐Aldrich) + protease inhibitor 2/3 1 : 100 each (Sigma‐Aldrich); used CERII per sample: 2.8 µL; and used NER per sample: 25 µL + phosphatase inhibitor 1 : 25 (Sigma‐Aldrich) + protease inhibitor 2/3 1 : 100 each (Sigma‐Aldrich).

Protein concentration was measured using the Pierce BCA Protein Assay Kit (Thermo Fisher Scientific) according to the manufacturer's instructions, and equal amounts of all samples were loaded onto a 4–12% SDS/PAGE (Bio‐Rad, Hercules, CA, USA). Following separation, proteins were transferred onto a PVDF membrane (GE Healthcare, Chicago, IL, USA). The membrane was blocked with TBST + 5% BSA (Sigma‐Aldrich), and the following antibodies were used for overnight incubation at 4 °C: p65 (1 : 1000, NF‐κB p65 D14E12 XP^®^ Rabbit mAb; Cell Signaling Technology, Cambridge, UK), p50 (1 : 1000, NF‐κB1 p105/p50 D4P4D Rabbit mAb; Cell Signaling Technology), c‐Rel (1 : 1000, c‐Rel D3B8S Rabbit mAb), IκB‐α (1 : 1000, IκBα 44D4 Rabbit mAb; Cell Signaling Technology), Pan‐Actin (1 : 1000, Pan‐Actin D18C11 Rabbit mAb; Cell Signaling Technology), and Histone (1 : 2000, Histone H3 D1H2 XP^®^ Rabbit mAb; Cell Signaling Technology). Protein bands were visualized using HRP‐linked anti‐rabbit antibody (1 : 20 000; Cell Signaling Technology) and SuperSignal West Pico Chemiluminescent Substrate (Thermo Fisher Scientific).

### *In vitro* Treg induction of transgenic T cells

2 × 10^7^ CD4^+^ T cells were transduced to overexpress either an empty control vector or the IκBmut construct (see above), both harboring an IRES‐CFP site. Four days after transduction, cells were FACS‐sorted for CFP expression and further used for Treg induction. For that purpose, FACS‐sorted cells from both control‐ and IκBmut‐transduced cells were treated with 100 U·mL^−1^ IL‐2 alone or 100 U·mL^−1^ IL‐2 + 100 nm rapamycin and stimulated with anti‐CD3/anti‐CD28 microbeads (cells : beads = 2 : 1). After 4 days of Treg induction, cells were either harvested for phenotypic and functional experiments or further cultured with 100 U·mL^−1^ IL‐2 in rapamycin‐free medium for another 4 days and used for iTreg suppression assays.

### Measurement of the activation status of co‐tg and FOXP3‐tg cells after pharmacological NF‐κB blockade with SC75741 (SC7)

Human CD4^+^ T cells were transduced either with an empty control vector (co‐tg) or a FOXP3‐encoding vector (FOXP3‐tg), both harboring an IRES‐GFP site. After transduction, cells were FACS‐sorted for GFP expression and activated with anti‐CD3/anti‐CD28 microbeads (cells : beads = 2 : 1) in the absence or presence of the NF‐κB inhibitor SC75741 (1.875 µm, 3.75 µm, or 7.5 µm; Selleckchem, Houston, TX, USA). After 24 h, surface expression of the early activation marker CD69 was measured by FACS analysis. Expression levels of SC7 treated co‐tg and FOXP3‐tg cells were normalized to those of co‐tg and FOXP3‐tg cells activated without SC7, respectively.

### Pharmacological blockade of NF‐κB signaling in Treg

Human PB Treg were FACS‐sorted as described above. Directly after sorting, 2 × 10^5^ Treg were incubated with either 100 U·mL^−1^ IL‐2 alone or 100 U·mL^−1^ IL‐2 + 3.75 µm SC75741 (Selleckchem). After 1‐h pre‐incubation, cells were stimulated with anti‐CD3/anti‐CD28 microbeads (cells : beads = 2 : 1). After 4 days of culture, cells were extensively washed in SC7‐free medium, FACS‐sorted for viable cells, and used for further coculture experiments.

### Genetic blockade of NF‐κB signaling in Treg

Human PB Treg were FACS‐sorted as described above. Directly after sorting, at least 5 × 10^5^ Treg were stimulated for transduction. After 2 days, cells were transduced with either an empty control vector or the IκBmut construct, both harboring an IRES‐CFP site. Four days after transduction, cells were sorted for CFP expression and used for further coculture experiments.

### Bisulfite conversion and FOXP3‐TSDR demethylation

For bisulfite conversion, genomic DNA from at least 2 × 10^5^ T cells was isolated with the Quick‐DNA Miniprep Plus Kit (Zymo Research, Irvine, CA, USA) according to the manufacturer's instructions. Then, 200–500 ng of isolated DNA was used for bisulfite conversion with the EZ DNA Methylation Kit (Zymo Research), which converts demethylated cytosine residues into uracil residues, while methylated cytosine residues remain unaffected.

For measuring of FOXP3‐TSDR demethylation, at least 15 ng of bisulfite‐treated DNA was used for qPCR using methylation (adapted from Zhuo *et al*. [42])‐ and demethylation‐specific primers.

FOXP3 methylation‐specific for: 5′ GGATAGGGTAGTTAGTTTTCGGAAC 3′
FOXP3 methylation‐specific rev: 5′ CGCCATTAACGTCATAACGA 3′
FOXP3 demethylation‐specific for: 5′ AGGATAGGGTAGTTAGTTTTTGGAAT 3′
FOXP3 demethylation‐specific rev: 5′ TTTTCCACCATTAACATCATAACAA 3′.

qPCR was performed with Luna^®^ Universal qPCR Master Mix (New England Biolabs) on a 7900HT Fast Real‐Time PCR system (Applied Biosystems) under standard cycling conditions.

Demethylation rate was calculated using the formula $100/[1+2^{\left({\text{Ct}}_{\text{dem}}-{\text{Ct}}_{\text{met}}\right)}]$ with Ct_dem_ representing the cycle threshold reached with demethylation‐specific primers and Ct_met_ representing the cycle threshold reached with methylation‐specific primers [42, 70, 71]. As quality control, genomic DNA from FACS‐sorted Teff and Treg was used.

### Coculture experiments with iTreg

Following iTreg polarization and in some experiments further culture (see above), viable cells were isolated by FACS sorting. CD4^+^ Tresp were labeled with a fluorescent cell proliferation dye (CPD – eFluor 670; eBioscience/Thermo Fisher Scientific). At least 5 × 10^4^ responder cells were cultured alone (stimulated and unstimulated) and in coculture with the sorted iTreg at a 1 : 1 ratio or the indicated ratios. Cells were activated with anti‐CD3/anti‐CD28 microbeads (responder cells : beads = 2 : 1). Proliferation of responder cells was measured 4 days after activation by flow cytometric analyses by gating on eFluor670^+^ cells only. The proliferation rate was calculated using the mean fluorescence intensity (MFI) of the eFluor670‐CPD and the following formula [72]:

$$\text{Division}\;\text{Index}=\log\left({\text{MFI}}_{\text{unstimulated}}/{\text{MFI}}_{\text{stimulated}}\right)/\log\left(2\right).$$

### Coculture experiments with PB Treg

For blockade of NF‐κB signaling in tTreg, see above. CD4^+^ Tresp were labeled with eFluor670‐CPD. 3 × 10^4^ Tresp were cultured alone (stimulated and unstimulated) and in coculture with the sorted, pretreated Treg at a 1 : 1 ratio. Cells were activated with anti‐CD3/anti‐CD28 microbeads (responder cells : beads = 2 : 1). Proliferation of responder cells was measured 4 days after activation by flow cytometric analyses by gating on eFluor670^+^ cells only. The proliferation rate was calculated using the MFI of the eFluor670‐CPD as described above.

### Statistical analyses

Data are represented as mean ± SD, if not otherwise stated, and were analyzed using graphpad prism (version 6; GraphPad Software, Inc., La Jolla, CA, USA). To assess differences between groups, paired *t*‐test or one‐way ANOVA was used, depending on the number of samples. Significance was defined according to the following *P*‐values: ns = not significant; *P ≤ 0.05; **P < 0.01; and ***P < 0.001.

## Conflict of interest

The authors declare no conflict of interest.

## Author contributions

LSZ performed cloning of the IκBmut construct and flow cytometric and functional analyses; MCG and RLJS contributed to cell isolation, cell culture, and flow cytometric analyses; DT and WFP assisted with thymidine incorporation assays and functional experiments; PS provided the cDNA library and reporter cell line and assisted with cloning and reporter assays; GE optimized bisulfite conversion protocols and measurement of demethylation; IS provided support with flow cytometric analyses and cell sorting; KGS coordinated the project and wrote the manuscript together with LSZ. All authors critically read the manuscript and contributed to the final formulation.
